# Influence of a changing wave climate on the quality and morphometry of the stalked barnacle *Pollicipes pollicipes* (Gmelin, 1789), along the coasts of NW Iberia

**DOI:** 10.1007/s11160-024-09838-2

**Published:** 2024-03-14

**Authors:** Raquel Peñas-Torramilans, Raquel Outeiral, José Santiago, Elsa Vázquez, Nicolas Weidberg

**Affiliations:** 1 CIM – Centro de Investigación Mariña and Departamento de Ecoloxía e Bioloxía Animal, Facultade de Ciencias do Mar, Universidade de Vigo, Vigo, Spain; 2 Confraría de Pescadores de A Guarda, Baixo Muro, 32, 36780 A Guardia, Galicia Spain; 3 Cofradía de Pescadores La Anunciada de Baiona, Casa del Mar, Segunda Planta, 36300 Baiona, Spain; 4 Department of Biological Sciences, University of South Carolina, Columbia, SC USA; 5 Present Address: Departament d’Enginyeria Civil i Ambiental (DECA), Laboratori d’Enginyeria Marítima (LIM), Universitat Politècnica de Catalunya - BarcelonaTech (UPC), C. Jordi Girona, 1-3, 08034 Barcelona, Catalunya Spain; 6 Present Address: Department of Organisms and Systems Biology, University of Oviedo, Oviedo, Spain

**Keywords:** Stalked barnacle, Wave climate, Resource quality, Ecomechanics, Coastal topography

## Abstract

Wave climate is shifting over the last decades along the Atlantic coasts of Europe ultimately driven by large-scale patterns of atmospheric variability forced by anthropogenic global warming. Changes in wave height and surf zone orbital currents are hypothesized to drive marked shifts in the shape of intertidal organisms such as the stalked barnacle *Pollicipes pollicipes*, whose quality and market price are known to decrease non linearly with the peduncle length: width ratio S. This study evaluates wave trends in NW Iberian Peninsula, using the Spanish Port System 2006–2020 SIMAR wave hindcast. On the other hand, trends in stalked barnacle morphology and quality are estimated from 26 sites at the management regions of Baiona and A Guarda between 2011 and 2020. Results show evidence of temporal changes in barnacle quality and, especially, morphometry caused by simultaneous shifts in winter wave induced orbital currents. Because of the non linear relationship between S and the high quality threshold, large increases in S are usually translated to small reductions in quality. However, we identified a tipping point around S = 2.4 that if surpassed can lead to great drops in barnacle quality. In addition, changes in wave forcing will have different effects at each extraction site, as trends in wave climate are decoupled from barnacle morphometry at steeper sites sheltered from the predominant wave direction. In conclusion, this knowledge could be applied to develop site specific barnacle harvesting strategies based on annual wave climate forecasts.

**Graphical abstract:**

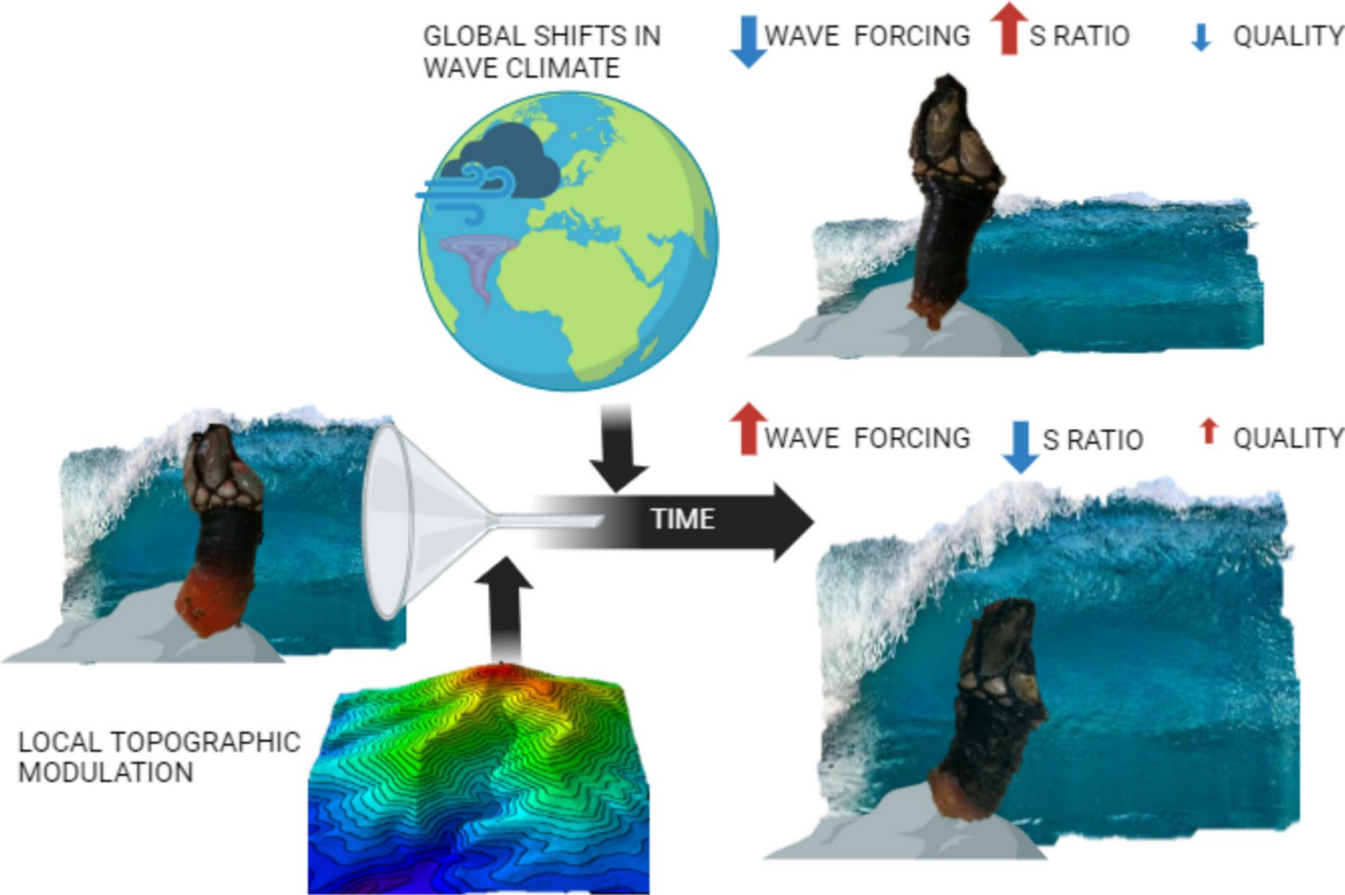

**Supplementary Information:**

The online version contains supplementary material available at 10.1007/s11160-024-09838-2.

## Introduction

Anthropogenic climate change due to the emissions of greenhouse gasses over the last decades has a major impact on the oceans (IPCC 2022). Besides showing contrasting trends in sea surface temperature (Lima and Wethey 2012), coastal areas suffer from extreme changes in wave climate (Bindoff et al. 2007; Denny et al. 2009; Wong et al. 2014). In this scenario, studying wave variations under climate change becomes crucial not only for building and protecting coastal infrastructures, but also to infer potential ongoing shifts in coastal ecosystems (Sierra and Casas-Prat 2014; Hewitt et al. 2015). Therefore, in recent years, the study of wave trends has been expanding both globally (Young et al. 2011; Reguero et al. 2019) and in the North Atlantic (Walden et al. 1970; Neu 1984; Draper 1986; Woolf et al. 2002; Shimura et al. 2013; Castelle et al. 2018). Analyses in the North Atlantic show a downward trend in wave height, but an upward trend in extreme wave height in the last decades for latitudes south of 55°N (Dupuis et al. 2006; Young et al. 2011). In contrast, trends north of 55°N seem to indicate increases in both wave height and waves in extreme events (Castelle et al. 2018; Reguero et al. 2019; Meucci et al. 2020). Wave variations may be linked to the evolution of climate indices, such as the North Atlantic Oscillation (NAO), which, under the influence of climate change, is increasingly in a positive phase for longer periods (Gillett et al. 2003). In the North Atlantic, when the NAO is positive (NAO+), the atmospheric pressure difference between the Icelandic low pressure and the Azores Anticyclone increases and moist westerly winds along the European Atlantic coasts intensify. In addition, winter storms move towards Northern Europe, resulting in warmer and wetter weather, associated with more frequent and intense storms, causing changes in wave patterns off the northern coasts of Spain (Hurrell 1995; Kushnir et al. 1995; Dodet et al. 2010; Feser et al. 2015). Nevertheless, more recent studies point to summer shifts in wave climate forced by enhanced tropical storms that may interact negatively with NAO forcing further north (Angus and Leckebusch 2020). These patterns reflect the complex nature of the link between global-scale changes and their consequences at regional and local scales (Orfila et al. 2005).

Climatic teleconnections impacting wave dynamics may have effects on shoreline biological communities, as these are highly susceptible to changes in the marine system. Marine ecomechanics focuses on the study of these interactions between mechanical impositions derived from the wave regime and the responses of organisms in their morphology and population structure (Denny 1988, 2014). Mainly, waves set unavoidable constraints on the growth and shape of organisms that scale to the level of entire populations (Denny 1988). Wave dynamics, usually translated to surf zone orbital currents, have been widely used in the field of ecomechanics to explain the distribution of mussels, corals, algae, and limpets across gradients of mechanical stress generated across and along the shore (Madin 2005; Denny and Gaylord 2010). For instance, ecomechanical models successfully explain the distribution limits of coral species with complex and elongated morphology across coral atolls (Madin 2005). These models are based on a simple ratio, S, which is the relative length of the organism with respect to its width. The higher the value of S, the greater the susceptibility of the organic structure to mechanical stress induced by orbital currents generated by breaking waves, and thus the greater the probability of break-up and increased mortality (Madin 2005).

In this context, European coasts are a host to a model organism for characterising changes in wave dynamics through an ecomechanical approach. The stalked barnacle, *Pollicipes pollicipes* (Gmelin 1791), is a crustacean distributed along the Atlantic coast of Europe and North Africa between 50°N (UK and France) and 14°N (Senegal) (Barnes 1996; Cruz et al. 2022). It can be found in the rocky intertidal zones exposed to strong wave action and forms dense aggregations in the most exposed areas (Barnes 1996; Borja et al. 2006). The morphology of this crustacean consists in a well-developed capitulum with calcified plates that protect the soft parts of the animal and a fleshy peduncle that attaches it to the rocky substrate. Its life cycle is biphasic, with a planktonic larval stage developing in the coastal ocean through 6 nauplius stages and a cyprid stage before recruiting to the adult habitat back in the rocky intertidal (Molares et al. 1994). Connectivity between isolated adult populations, as well as their total biomass and reproductive output, is highly dependent larval dispersal and the seasonal variability of the coastal upwelling process (Macho et al. 2005; Quinteiro et al. 2007; Campo et al. 2010; Rivera et al. 2013; Höfer et al. 2017; Román et al. 2022). Barnacles are a highly valued commercial species and are intensively harvested in Morocco, Spain, and Portugal (Boukaici et al. 2012; Parada et al. 2012; Sousa et al. 2013). In Galicia (northwest Spain), *P. pollicipes* is a resource of high economic and social value. Between 2010 and 2020 it reached an average price of 27 €/kg in Galician fish auctions, with maximums of more than 280 €/kg, obtaining a total average value of almost 9 million euros per year (www.pescadegalicia.gal access September 2023). In Galicia and northern Spain, this fishery is managed at the local level by harvesters’ associations (“cofradia”) (Rivera et al. 2014, 2016a). The harvesters distinguish barnacles into two groups according to their morphology: elongated barnacles, which have a lower market value, and standard barnacles with stubby, wide peduncles that are edible and highly valued (Parada et al. 2012). As a morphological index to classify barnacles, a polynomial function is used that depends on both the length and width of the individuals as well as on the criteria of the barnacle collectors (Parada et al. 2013). Such function could be a valuable tool to split barnacle stocks into good and bad quality categories, but its nature is not linear in relation to barnacle width and, in turn, to S (Parada et al. 2012), which implies that potential wave driven morphological changes may not be translated to shifts in quality in a straightforward way. Regardless of the exact relationship between subjective quality criteria and morphometrics, quality classifications have been applied not only to individual barnacles, but to specific sites on a very small spatial scale (Rivera et al. 2014, 2016b). Furthermore, the topographic characteristics of each extraction site along the coast match remarkably well the quality of the resource, with higher frequencies of wider animals at more exposed and wavy locations (Rivera et al. 2016a, b). Accordingly, key nearshore landscape metrics like bottom slope, orientation and complexity are known to regulate many different coastal processes, including larval dispersal, settlement and sediment transport and retention (Kamphuis et al. 1986; Wolanski and Spagnol 2000; Defeo and McLachlan 2011; Morgan et al. 2017; Shanks et al. 2017; Weidberg et al. 2018). Therefore, one could argue that if spatial variability in wave forcing regulates barnacle morphology along the coast, temporal variability in wave climate may also impact barnacle populations. Nevertheless, site specific morphometric temporal trends have not been studied yet for this species.

Taking into account the above, *Pollicipes pollicipes* seems to be a suitable species to apply ecomechanical theories to understand whether large-scale wave variations have an impact on barnacle communities at the local scale. The aim of this paper is to evaluate changes in stalked barnacle morphology and quality at the local scale, possibly influenced by wave trends along the Galician coast. Thus our objectives are: (1) to infer temporal trends in maximum wave heights and their corresponding orbital currents along the southern coast of Galicia; (2) to infer temporal trends in both *P. pollicipes* quality and morphology at multiple nearby coastal extraction sites and their mutual relationship and correspondence with changing wave forcing; and (3) to identify refractory (permeable) sites to the effects of wave dynamics on *P. pollicipes* morphology and quality and the topographic characteristics responsible of such spatial modulation.

## Methodology

### Study area

**Fig. 1:**
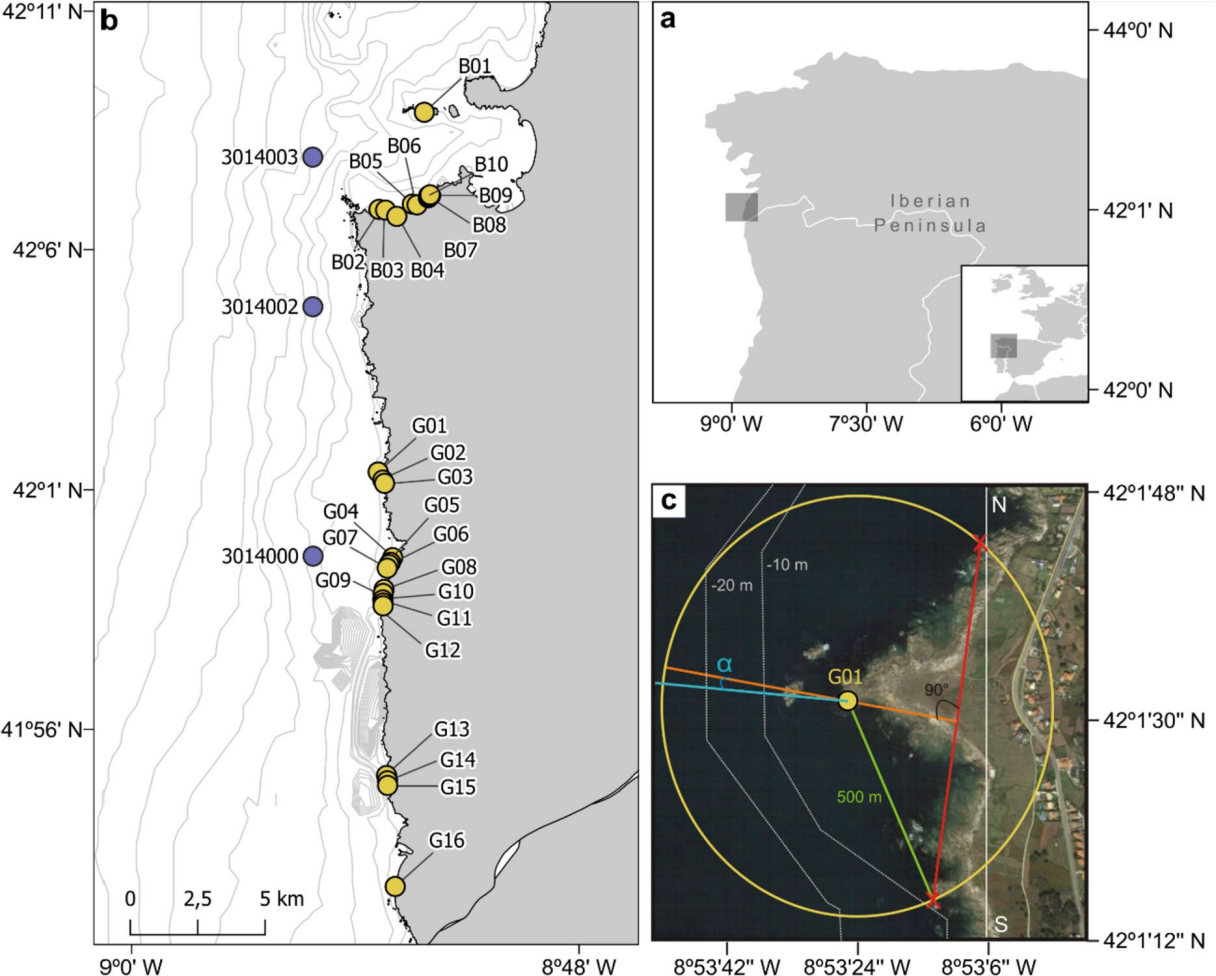
**a** General map of the Iberian Peninsula, in the northwest of the map the study area is marked. **b** Map of *Pollicipes pollicipes* extraction points in the areas of Baiona and A Guarda (yellow) and SIMAR wave points (purple). The grey lines show the bathymetry (10 m). **c** Detail of point G01 (Puntal Orelludas). A hypothetical circumference of 500 m radius is drawn from the point. The red crosses show the two points crossed by the circumference on the coastline. The orange line creates a 90° angle with the red line drawn between the two crosses and represents the axis perpendicular to the coast. The blue line is the direction of the swell and alpha is the absolute difference between the perpendicular axis and the angle of incidence of the swell at point G01

The study has been carried out in two areas of the Galician coast, Baiona and A Guarda, in Rias Baixas, in the northwest corner of the Iberian Peninsula (Fig. 1b). It is one of the most productive coastal areas of the world due to the Iberian Upwelling System, which stretches from Portugal to Spain and forms the northern boundary of the Iberian-North African upwelling system (Blanton et al. 1984). At these latitudes, coastal winds respond to the local forcing on synoptic time scales driven by sea-land thermal breezes and to a mesoscale forcing exerted by the Azores Anticyclone (Sousa et al. 2019). In the upwelling season, from March–April to September–October, northerly winds predominate. In the subsidence season, which occurs during the rest of the year, southerly winds produce coastal downwelling (Wooster et al. 1976; McClain et al. 1986; Bakun and Nelson 1991). Nevertheless, to the south of Cape Finisterre and especially off Rias Baixas, upwelling can occur in winter (Álvarez et al. 2009). These temporal patterns in wind dynamics are translated to waves, with wind waves with shorter period and heights in summer. In winter, besides the wind waves caused by local low pressure systems coming from the Atlantic, large swell arrives from eastward storms passing through the British Islands (Dupuis et al. 2006; Dodet el al. 2010; Castelle et al. 2017).

### Wave time series

Wave time series in this study are derived from the SIMAR dataset provided by Puertos del Estado (www.puertos.es). The historical SIMAR data are built on wave and wind time series obtained from numerical modelling of the ocean, provided by Puertos del Estado, and of the atmosphere, carried out by the Agencia Estatal de Meteorología (AEMET, www.aemet.es). The subset used is WANA, which provides information from 2006 to the present. The WANA series are periodically upgraded, so that the spatial and temporal resolution has been changing. For the wind data, the data come from the HIRLAM (until 2018) and HARMONIE-AROME models (2018 onwards), which increased the temporal resolution from 6 to 1 h, and the spatial resolution from 30 to 2.5 km. For the wave model, the WAM and WaveWatch models fed by the AEMET wind fields are used, with a spatial resolution of 2.8 km in the Iberian Peninsula.

The wave series of 3 SIMAR points, 3014000, 3014002 and 314003 (Fig. 1a) were analysed and the maximum inshore wave height (*H*_*i*(*max*)_) and orbital current (*u*) were calculated. *H*_*i*(*max*)_ was calculated from the offshore significant height (*H*_*o*_), choosing the maximum daily wave height in metres (m) from hourly significant heights considering shoaling effects produced by the proximity of the coast. The depth of SIMAR points was calculated using SeaDAS software (8.0.0) with GEBCO (General Bathymetric Chart of the Oceans) bathymetry (GEBCO 2020), with resolution 15 s of degree. The *H*_*i*_ and *u* calculations were made with the following formulas proposed by Denny (1995):

1 $$H_{i(max)} = \left\{ {\frac{{H_o (\sinh \left( {2k_i d_i } \right)[2k_o d_o + \sinh (2k_o d_o )])}}{{\sinh \left( {2k_o d_o } \right)[2k_i d_o + {\text{sinh}}(2k_i d_i )])}}\cdot \frac{{\tanh \left( {k_o d_o } \right)}}{{\tanh \left( {k_i d_i } \right)}} } \right\}^{1/2}$$

where *d*_*o*_ and *d*_*i*_ are the offshore and inshore depths respectively, being *d*_*o*_ the depth where the SIMAR point is located and *d*_*i*_ the depth of the shallowest intertidal zone (1 m). In addition, *k*_*o*_ and *k*_*i*_ are the offshore and inshore wave numbers respectively. And the wave number is as presented in the following formula:

2 $$k = \left( {\frac{2\pi }{{l_w }}} \right)$$

The wavelength *l*_*w*_ given a local depth *d*, can be calculated with the following expression:

3 $$l_w = \left( {\frac{gT_p^2 }{{2\pi }}} \right) \left( {\tanh \left[ {\frac{4\pi^2 d}{{T_p^2 g}}} \right]} \right)^{1/2}$$

where *g* is gravity (9.81 m/s^2^) and *T*_*p*_ is the wave period.

Finally, the orbital currents for maximum inshore daily wave heights (*u*_*i*(*max*)_) can be calculated with all the previous parameters following this equation:

4 $$u_{i(max)} = [gH_{i\left( {max} \right)} ]^{1/2}$$

For each SIMAR point, a time series analysis was applied to determine the long-term trend and to obtain a slope or rate of change over time for *H*_*i*(*max*)_ and *u*. The time series analysis was performed using a Generalised Additive Mixed Model (GAMM) (Chen 2000; Wood 2006) to analyse, on the one hand, the seasonal component of the time series and, on the other hand, the long-term trend component. The number of knots for the smoothing splines of the long term and seasonal components was 15 and 12, respectively, as used in previous works with GAM analyses applied to time series of similar lengths (Weidberg et al. 2020). A linear fit was applied to the long term component to obtain a linear slope. These models are robust to discontinuous series or irregular time intervals along the time series (Simpson 2018). The analyses were run with the "mgcv" package (Wood 2006) for R statistical software version 4.2.2 (R Core Team 2022). Furthermore, annual variation of *u*_*i*(*max*)_ were also plotted for each point considering only the three consecutive months of strongest and weakest *u*_*i*(*max*)_ means (January, February, and March; June, July, and August) by using linear fits.

### Morphometry and quality of *Pollicipes pollicipes*

*Pollicipes pollicipes* morphology can be estimated from the following formula proposed by Madin (2005):

5 $$S = \frac{TL}{{DBC}}$$

This is a biometric index calculated from the total length of the barnacle (TL) and the diameter of the base of the capitulum (DBC) (Parada et al. 2012). Therefore, an organism longer than wider will have a morphological index greater than 1 while organisms wider than longer will have a morphological index less than 1. This index has previously been applied for sessile benthic organisms and is a measure related to the mechanical stress forced by currents that the animal is able to resist before breaking. Thus, the higher the S the more susceptible the organism will be to breaking and detaching from the substrate (Denny 1995). On the other hand, according to Parada et al. (2012) the different *P. pollicipes* quality typologies are related to TL and DBC, but following a non-linear relationship, as they follow the sigmoidal functions shown in Eq. 6, for individuals measured after thawing.

6 $$TL = 67.8055 + \frac{(10.9002 - 67.8055)}{{1 + \left( {\frac{DBC}{{12.216}}} \right)^{2.8008} }}$$

For this study, data came from two different management plans: the Cofradía de Pescadores de 'Santa Tecla' de A Guarda and the Cofradía de Pescadores 'la Anunciada de Baiona'. Harvesters’ associations provided TL and DBC yearly data between 2011 and 2020, which were used to analyse the variations in the morphological index over time. Transects were performed across the intertidal strip and for each level (upper, middle, and lower); between 30 and 50 individuals were gathered at various sites, usually from the same group to obtain a representation of all size classes in the population. If groups were not found because cover was low, isolated individuals were collected. The collected barnacles were then frozen, and TL and DBC were measured for all individuals with a DBC > 2 mm. A total of 16 sites in A Guarda and 10 in Baiona with more than 6 yearly samplings were studied (Fig. 1a and Table 1).

**Table 1:** List of *Pollicipes pollicipes* extraction points within the fishing guilds of Baiona and A Guarda

Point code	Point name	Coordinates	Slope (m)	α (**°**)	Q	Time series
*Baiona*						
B01	Longueirón	42.14618, − 8.86864	− 0.029	76.18	VH	2014–2020
B02	Baleal	42.11376, − 8.88905	− 0.108	74.57	H	2015–2020
B03	Baleal	42.11351, − 8.88607	− 0.038	78.18	H	2014–2020
B04	As Vacas	42.11143, − 8.88105	− 0.05	86.49	VL	2015–2020
B05	As Vacas	42.11553, − 8.87406	− 0.042	67.63	VL	2014–2020
B06	As Vacas	42.11525, − 8.87196	− 0.036	57.02	VL	2014–2020
B07	Bombardeira	42.11763, − 8.86688	− 0.015	49.58	H	2014–2020
B08	Bombardeira	42.11829, − 8.86675	− 0.016	50.94	H	2014–2020
B09	Bombardeira	42.11840, − 8.86583	− 0.02	44.06	H	2014–2020
B10	Bombardeira	42.11861, − 8.86611	− 0.02	45.42	H	2014–2020
*A Guarda*						
G01	Puntal Orelludas	42.02602, − 8.88958	− 0.047	0.98	VH	2011–2020
G02	A Rachada	42.0233, − 8.887471	− 0.026	9.05	H	2011–2020
G03	Terra Castriños	42.02222, − 8.88672	− 0.028	21.65	H	2011–2020
G04	N Isliñas	41.99739, − 8.88310	− 0.063	12.45	M	2011–2018
G05	N Bombardeira	41.99582, − 8.88408	− 0.009	10.68	M	2011–2018
G06	Frente Bombardeira	41.99509, − 8.88476	− 0.095	3.12	M	2011–2018
G07	Orja	41.99388, − 8.88556	− 0.015	0.38	VH	2013–2020
G08	Os Piringallos	41.98677, − 8.88699	− 0.091	5.67	VL	2011–2018
G09	Regueiriño do torto	41.98561, − 8.89071	− 0.091	3.22	VL	2013–2018
G10	Homiño	41.98327, − 8.88757	− 0.016	12.87	L	2011–2019
G11	Frente Agoeiriña	41.98224, − 8.88740	− 0.018	12.2	L	2014–2019
G12	Puntal Coraná	41.98131, − 8.88742	− 0.023	10.56	M	2013–2018
G13	S Carabeleira	41.92461, − 8.88598	− 0.057	18.74	VL	2013–2019
G14	N Os Soles	41.92284, − 8.88557	− 0.056	17.92	VL	2011–2019
G15	S Os Soles	41.92128, − 8.88550	− 0.104	16.91	VL	2011–2019
G16	Frente Depuradora	41.88751, − 8.88218	− 0.011	11.43	VH	2011–2020

The following information is shown for each site: point code, point name, location, slope, absolute difference in degrees between the angle of wave incidence and the axis perpendicular to the coast (α) and quality index of each site (Q; VH = Very high, H = High, M = Medium, L = Low, VL = Very low)

For *P. pollicipes* quality, for each site, intertidal level and sampling time, the percentage of good quality individuals or high quality fraction (HQF) was calculated with Eq. 6, using the curve as a threshold to separate good from bad quality individuals.

Besides our quality assessment based on Parada’s equation, a subjective evaluation of barnacle quality according to harvesters’ experience at each site was carried out in 2009 with the aid of the technical assistants of the 2 management plans. From this evaluation 5 quality categories arose: very high, high, medium, low and very low. We compared this categorization with our HQF estimates using a GLMM with a Gamma distribution using the quality categories as a factor.

### Topographic variables

To calculate the topographic site specific variables, a circle of 1000 m of diameter was drawn around each barnacle extraction site (Fig. 1c). The diameter of the circle was chosen to capture the main interactive effects between nearshore topography and wave forcing (Weidberg et al. 2018). Then, to get a proxy of the coastline orientation around the sites, a line was drawn between the 2 coastline-circle intersection points (Rivera et al. 2016a, b). Thus, the absolute difference in degrees between the angle of wave incidence and the axis perpendicular to the coastline (α) was calculated (Fig. 1c and Table 1). The mean wave incidence angle was calculated for each SIMAR point from the 2006 to 2020 time series of wave direction using circular statistics (library *CircStats* in R, Lund and Agostinelli 2001) and then these means were assigned to the closest barnacle extraction sites. On the other hand, along the 1000 m perpendicular lines, depths were obtained from the GEBCO bathymetry. The nearshore slope was then approximated as the linear slope of the regression between depth and distance.

### Temporal patterns

To infer if barnacle morphometry and quality differed between sites, times, and positions across the intertidal, S and HQF were submitted to Generalised Linear Mixed Models (GLMMs) with Site and Intertidal Levels as factors and Time as continuous predictor. All sites from the 2 fishing guilds with at least 6 consecutive sampling years from 2011 to 2020 were considered (26 sites in total). Three level of the factor Intertidal Level (lower, medium, upper) were used. As both S and HQF datasets presented highly skewed, non-normal distributions, GLMMs based on Gamma distributions with logarithms as link functions were used. For HQF it was not possible to build a full factorial design, as there was not any replication for the second order interaction (Site*Intertidal Level*Time) because only a single HQF value for the population can be obtained at this level. All possible models were obtained from the GLMMs and then ranked by the conditional Akaike Information Criterion AICc using the function “dredge”, which penalises model complexity while favouring the amount of variability explained. The analyses were run with the "MuMIn" package (Bartoń 2022) for R statistical software version 4.2.2 (R Core Team 2022). McFadden’s pseudo R^2^ for the best models was provided as a proxy of variability explained (McFadden 1974).

In addition, we obtained long-term trends of both S and HQF from the linear slopes of those 2 variables with time. Since the polynomial function defining the quality threshold as perceived by barnacle collectors are related to S, a correlation between the linear slopes of HQF and S was performed to check whether morphometric changes imply observable changes in the quality of the resource.

### Morphology – wave stress coupling

To infer if there was an association between wave dynamics and trends in *P. pollicipes* morphometry, mean currents were calculated for the *u*_*i*(*max*)_ peaking months (January, February, March) preceding each sampling from the closest SIMAR point. Mean S and HQF were calculated pooling all three intertidal levels together for each date. Then, the two variables were regressed against mean *u*_*i*(*max*)_ for the 2 management plans and for each of the 26 sites individually to inspect how the degree of coupling between barnacle morphometry/quality and wave stress varied in space.

The determination coefficients R^2^ for each site and variable (R^2^S and R^2^HQF for S and HQF, respectively) were further used to perform GLMMs using relative angle of incidence (α), mean orbital current for each specific site (*ū*) and shoreline slope (slope) as continuous predictors. After checking the data distributions for both R^2^S and R^2^HQF, this time we assumed normal distributions to perform the GLMMs. Again, all possible models were ranked by the AICc using the function “dredge”. The analyses were run with the "MuMIn" package (Bartoń 2022) for R statistical software version 4.2.2 (R Core Team 2022). The goodness of fit of the best models (R^2^) was also provided. With these procedures we want to infer the topographic characteristics driving the degree of coupling between wave dynamics and barnacle morphometry. We expect to find higher degrees of coupling at exposed sites perpendicular to the prevalent wave direction, with strong orbital currents and gentle slopes where waves can entrain easily.

### Quality vs morphometry in relation with Parada’s limit

In addition to the comparison of long term trends in quality and morphometry, we also inspected the relationship between S and HQF for a given population at a certain time. Thus, we regressed mean HQF yearly values from all sites against their corresponding mean S using sigmoidal fits. In this way it is possible to quantify the change in quality for a given change in morphometry.

To better understand the consequences of the observed changes in S in our populations, we represented mean, maximum and minimum S yearly means considering all sites and years in the TL-DBC plot together with Parada’s limit for quality assessment (Eq. 6). With this representation it is possible to infer the range in size (DBC) that corresponds to good quality barnacles depending on real and representative S means.

## Results

**Fig. 2:**
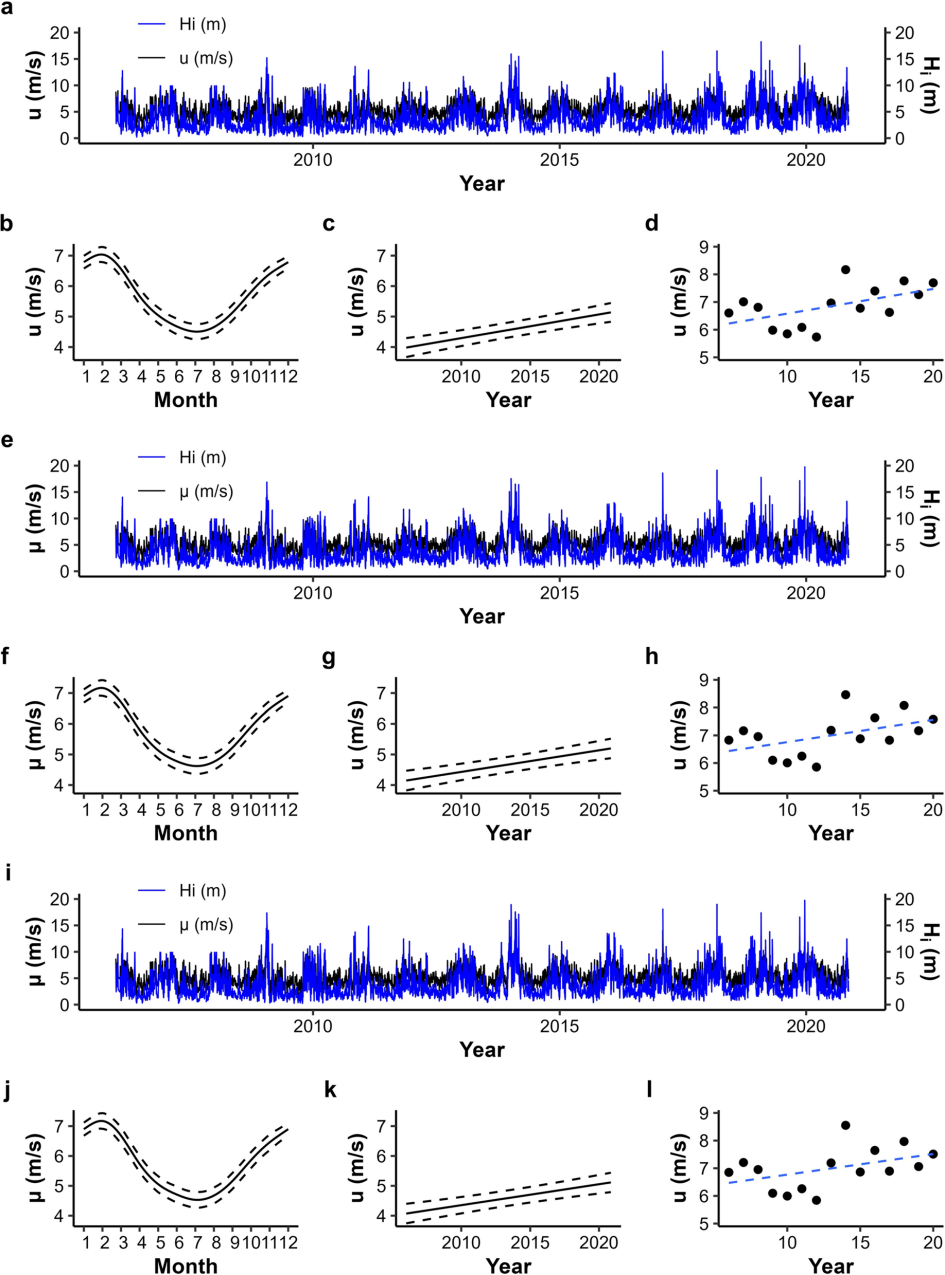
Time series between 2006 and 2020 of *H*_*i*(*max*)_ and u of SIMAR points 3014000, 3014002 and 3014003 (**a**, **e**, **i** respectively). Seasonal variation (**b**, **f**, **j**) and annual trend of *u* (**c**, **g**, **k**). Annual variation of *u* considering only the three consecutive months of strongest *u* (January, February, and March) (**d**, **h**, **l**)

Time series of daily *H*_*i*(*max*)_ and *u* for the 3 SIMAR locations clearly show a high frequency variability superimposed to seasonal fluctuations and long term increases (Fig. 2a, e, i). Highly significant (*p* < 0.0001) increments between 0.070 m*s^−1^*year^−1^ and 0.078 m*s^−1^*year^−1^ in µ were detected by the GAM analyses (Fig. 2c, g, k). The seasonal pattern was also found to be significant (*p* < 0.0001), peaking between January and March (Fig. 2b, f, j). When yearly means for this period of maximum orbital currents are represented along time, the positive trend still appears evident (between 0.075 m*s^−1^*year^−1^ and 0.089 m*s^−1^*year^−1^) but much less pronounced and only slightly significant at SIMAR’s southernmost 301400 point, *p* = 0.035 (Fig. 2d, h, l). In fact, such increase disappears if the period when most of the *P. pollicipes* monitoring samplings were carried out from 2014 onwards (Table 1) is considered. Trends are much clearer in summer, with highly significant increments around 0.07 m*s^−1^*year^−1^ and *p* < 0.005 for the 3 SIMAR locations (see Figure 1 in Supplementary Material).

Topographic variables changed between sites. The relative incidence angle α changed from 0.9° to 86°, being consistently larger along the coast of Baiona, as this nearshore area faces north while waves usually comes from the west (Table 1). Regarding nearshore slopes, these ranged from − 0.009 to − 0.1. There is not a consistent spatial aggregation of steeper or gentler sites (Table 1).

**Fig. 3:**
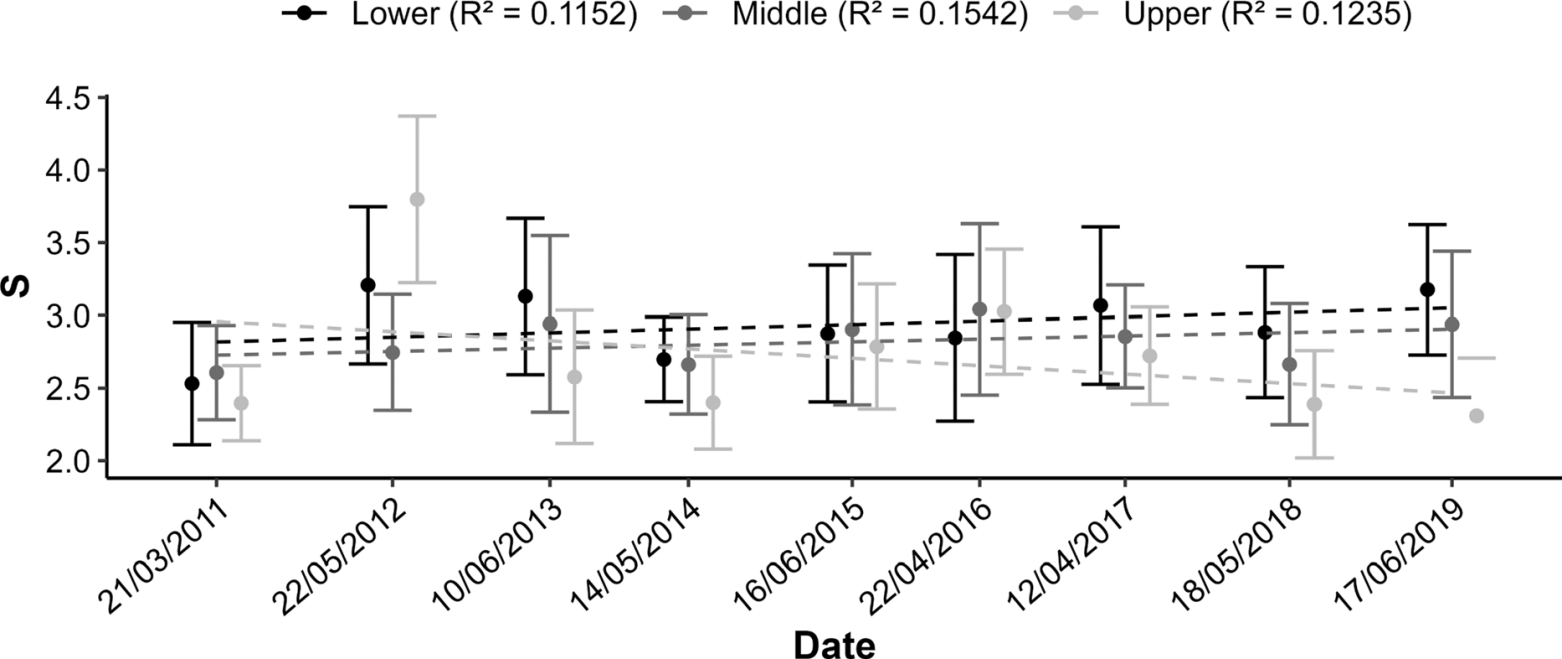
Example of variations of the morphological index S over the years at site G10 at lower, middle, and upper intertidal levels

To model the change in morphological index S over time, considering intertidal level and site, the GLMM with the lowest AIC classification was selected. In this case it was the first ranked model that included site, level, and date with all their interactions (AIC = 31,544.8) (Table 2, see Table 1 in Supplementary Material). McFadden’s pseudo R^2^ for this model is 0.21. Thus, morphological trends in time changed among sites and intertidal levels. Overall, at a significance level of *p* = 0.05, 27 out of a total of 78 time series at different sites and intertidal levels showed significant morphological trends towards relatively more elongated individuals, 12 significant trends to relatively wider barnacles and 39 non-significant trends. Figure 3 shows the example of site G10, where increases in the morphological index S are recorded for the upper and middle level and a decrease for the lower level. Although time is significant in all 5 best models, its interactive effects with intertidal level and site are also included in all models, thus preventing any general common temporal trend in S for all sites (Table 2).

**Table 2:** List of the five best models resulting from the GLMM analyses for the morphological index S taking into account the time, the intertidal level and the site

(Int)	TIME	LEVEL	SITE	TIME:LEVEL	TIME:SITE	LEVEL:SITE	TIME:LEVEL:SITE	*df*	logLik	AICc	Delta	Weight
0.0512	1.69E−05	+	+	+	+	+	+	157	− 15,614.4	31,544.8	0	1
0.4877	6.54E−06	+	+		+	+		105	− 15,760.8	31,732.5	187.63	0
0.4934	6.41E−06	+	+	+	+	+		107	− 15,760.1	31,735.2	190.36	0
0.5266	6.32E−06	+	+	+	+			57	− 16,006.8	32,127.8	582.96	0
0.5074	6.78E−06	+	+		+			55	− 16,011.5	32,133.3	588.46	0

**Fig. 4:**
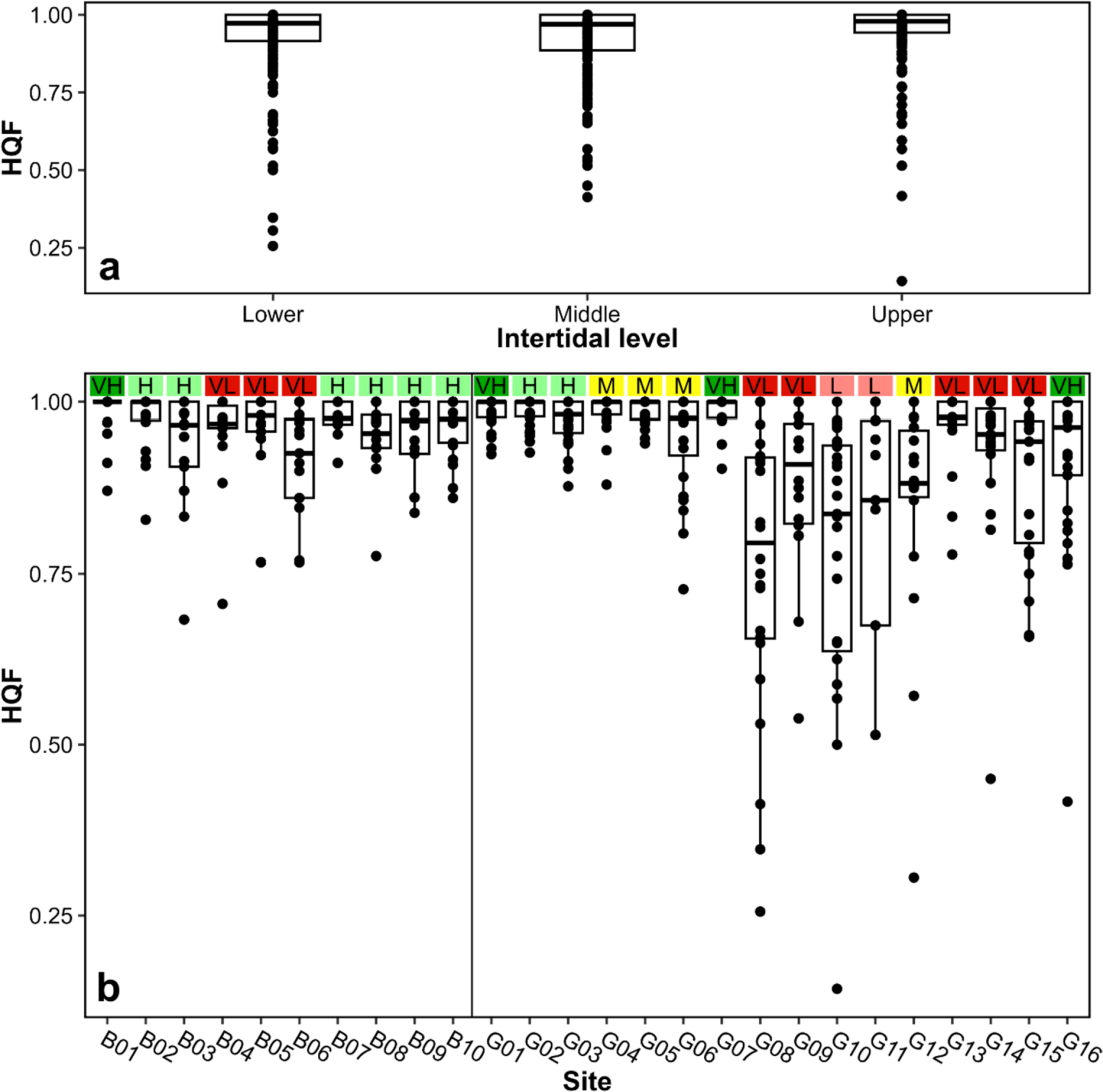
Boxplots of High Quality Fraction (HQF) between the different intertidal levels (**a**) and sites (**b**) of A Guarda and Baiona. The black points correspond to each yearly sampling, the black lines show the median and the boxes represent the interquartile range. Quality index of each site: VH = Very high, H = High, M = Medium, L = Low, VL = Very low

The comparison between the 5 quality categories established by the harvesters and our HQF shows a significant correspondence (*p* < 0.01) but with a complex association. Overall, HQF means for sites with medium, high and very high quality do not differ significantly, and they are significantly higher than those for low quality sites. However, very low quality sites present HQF means significantly higher than those in low quality sites and do not differ from medium quality sites (see Figure 2 and Table 2 in Supplementary Material). On the other hand, the best model to explain HQF patterns includes level and site without any interaction between factors (AIC = − 759) (Table 3, see Table 3 in Supplementary Material). Figure 4 shows slightly different HQF values between the different intertidal levels with a high variability. Overall, HQF is significantly higher within the upper intertidal level. When HQF is compared among sites, lower mean HQFs characterise the southern sites of A Guarda from G08 to G12. Time becomes significant in 3 out of the 5 best models, showing a negative relationship with HQF, thus pointing to a long term trend reduction (Table 3). McFadden’s pseudo R^2^ for this model is 0.23.

**Table 3:** List of the five best models resulting from the GLMM analyses for HQF considering the date, the intertidal level and the site

(Int)	DATE	LEVEL	SITE	DATE:LEVEL	DATE:SITE	LEVEL:SITE	*df*	logLik	AICc	Delta	Weight
− 0.02421		+	+				29	410.02	− 759	0	0.401
0.3059	− 7.80E−06	+	+				30	410.755	− 758.2	0.75	0.276
− 0.01339			+				27	406.902	− 757.2	1.83	0.161
0.3201	− 7.88E−06		+				28	407.645	− 756.4	2.54	0.112
0.06224	− 2.06E−06	+	+	+			32	411.285	− 754.8	4.15	0.05

**Fig. 5:**
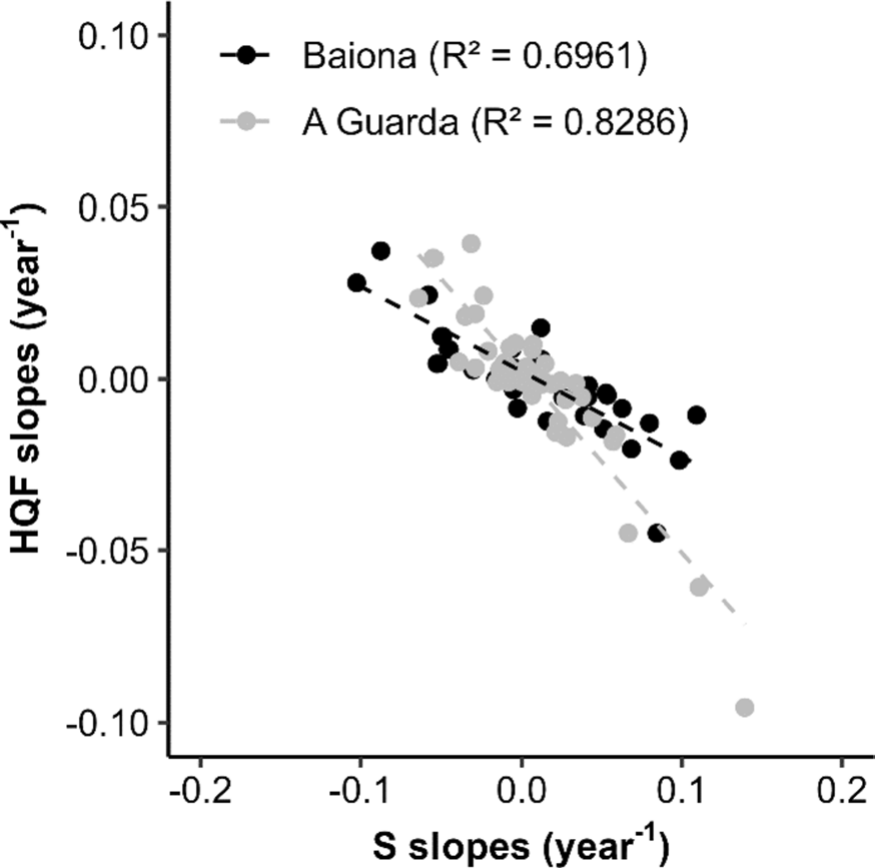
Comparison between the trends of the morphological index (S) and the high-quality fraction (HQF) of all sampling sites from Baiona (black points) and A Guarda (grey points). R^2^ = regression coefficient

When temporal trends in S were regressed against temporal trends in HQF a significant negative relationship was obtained. Such relationship was stronger in A Guarda, with a variance explained of 69.61% in HQF trends in Baiona (*p* < 0.01) and 82.86% in A Guarda (*p* < 0.0001). This negative relationship indicates that increasing/decreasing temporal trends in S (relative peduncle elongation/shortening) are translated to significant decreases/increases in HQF along time (Fig. 5).

**Fig. 6:**
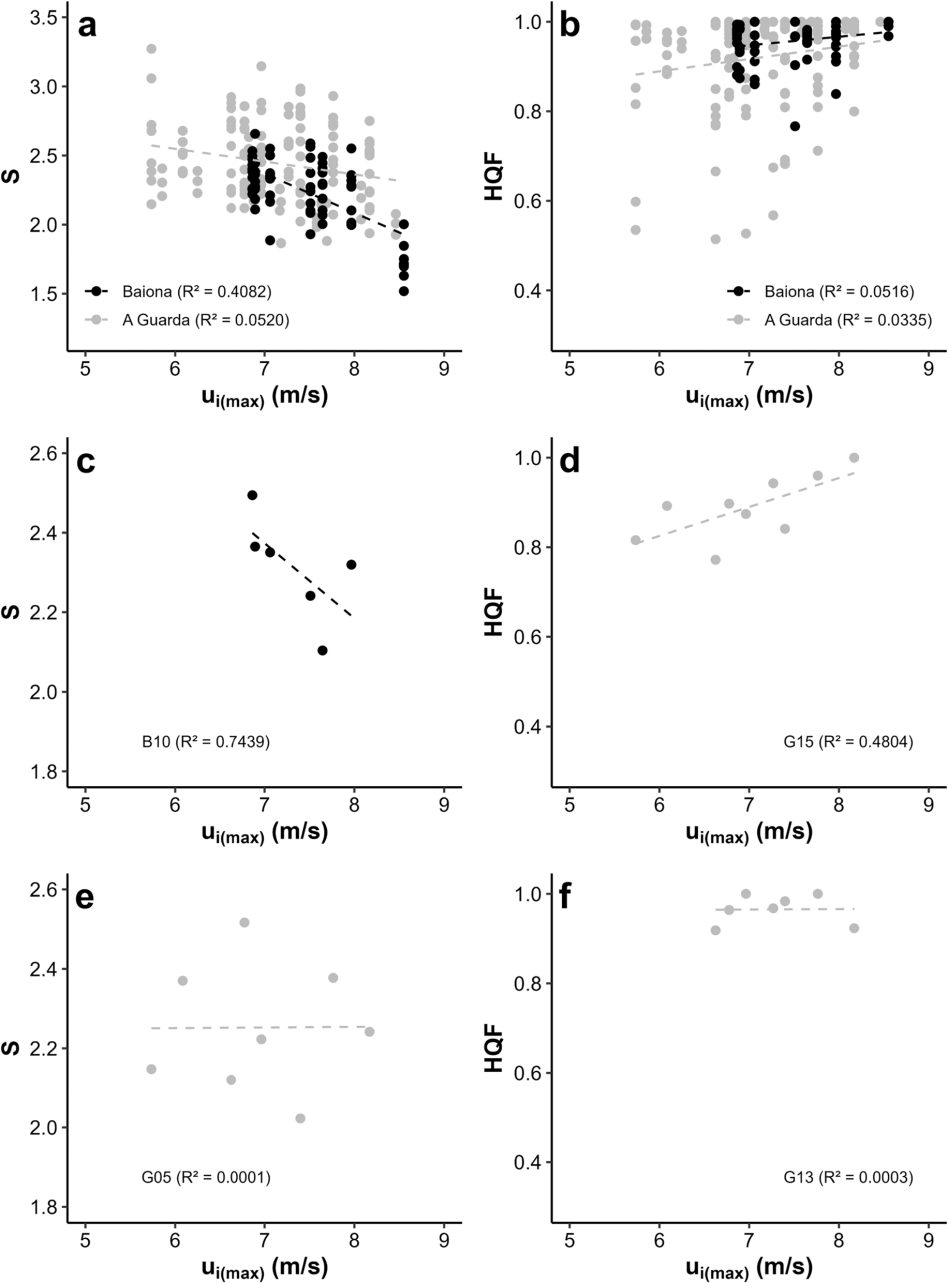
Variability among sites in the degree of coupling between *u*_*i*(*max*)_ and HQF (**a**, **c**, **e**) and between *u*_*i*(*max*)_ and S (**b**, **d**, **f**). Linear regression are shown for the 2 fishing guilds (**a**, **b**), and for those sites exhibiting the best (**c**, **d**) and worst (**e**, **f**) fits. Grey and black points represent the datasets for A Guarda and Baiona, respectively. R^2^ values are shown in parentheses

The linear regressions between S and u means for each site and year point to varying, site-specific degrees of coupling between orbital currents and *P. pollicipes* morphometrics (Fig. 6). Overall, a better negative fit is obtained for Baiona with respect to A Guarda, which indicates that relative barnacle width increases with u (Fig. 6a). Site specific goodness of fits range from 75% at B10 to only 0.01% at G05 (Fig. 6c, e). Similar results were obtained for HQF means, although their positive relationship with u is much weaker and only significant for A Guarda (*p* = 0.035, Fig. 6b). Nonetheless, at the site specific level, R^2^ values reach 48% at G15, while the lowest is obtained at G13 (Fig. 6d, f). Thus, the way in which increments in orbital currents are translated to higher percentages of high quality barnacles vary among sites.

**Fig. 7:**
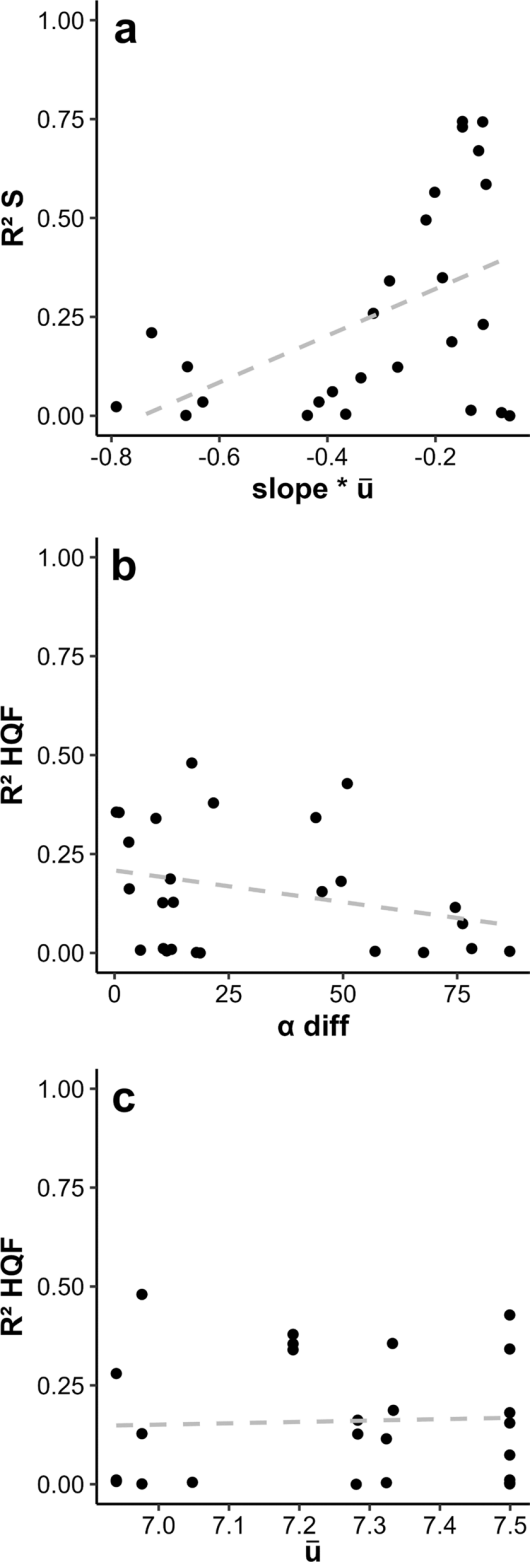
Linear regressions between R^2^S (**a**) and R^2^HQF (**b**, **c**) and the predictors selected in the best GLMMs: the interaction slope * $\overline{u}$ (**a**), the incidence angle (**b**) and the average orbital current $\overline{u}$ (**c**)

According to the GLMM results, the site-specific degree of coupling between S and *u* depends in great extent on the interaction between nearshore slope and $\overline{u}$, as this term becomes significant in 4 out of the best 5 models (Table 4). In essence, R^2^S increases with the combined of effect of gentler slopes and reduced orbital currents (Fig. 7a, Table 4, see Table 4 in Supplementary Material). The best model explained up to a 58% of the variability explained. When examined in models without their corresponding interactions, reduced slopes, small α angles and stronger mean orbital currents are associated with higher R^2^S values (Table 4).

**Table 4:** List of the five best models resulting from the GLMM analyses for the degree of coupling between S and µ (R^2^S) with relative incidence angle (α), average orbital current (¯$\overline{u}$) and coastal slope as predictors

(Int)	α	SLOPE	$\overline{u}$	α: SLOPE	α:$\overline{u}$	SLOPE:$\overline{u}$	α: SLOPE:$\overline{u}$	*df*	logLik	AICc	delta	weight
− 9.631		− 144.3	1.386			20.58		5	10.9	− 8.7	0	0.773
− 9.660	− 5.65E−05	− 144.0	1.390			20.54		6	10.9	− 5.3	3.42	0.140
− 10.700	− 1.83E−03	− 166.4	1.541	− 0.0360		23.79		7	11.1	− 2.0	6.74	0.027
− 10.720	4.02E−02	− 152.1	1.538		− 0.0054	21.68		7	11.0	− 1.7	7.04	0.023
− 3.745		2.766	0.568					4	5.13	− 0.4	8.36	0.012

Regarding R^2^HQF, the best GLMM points to a model with α and $\overline{u}$ as the the best fit, although with a modest R^2^ of 12%. In particular, as α increases R^2^HQF decreases α. In fact, this angle was significant in 4 out of the best 5 models. On the other hand, although it appears within the best model, $\overline{u}$ presented a very weak, non-significant positive association with R^2^HQF (Fig. 7b, c, Table 5 and see Table 5 in Supplementary Material).

**Table 5:** List of the five best models resulting from the GLMM analyses for the degree of coupling between HQF and µ (R^2^HQF) with relative incidence angle (α), average orbital current (¯µ) and coastal slope as predictors

(Int)	α	SLOPE	$\overline{u}$	α: SLOPE	α:$\overline{u}$	SLOPE:$\overline{u}$	α: SLOPE:$\overline{u}$	*df*	logLik	AICc	Delta	Weight
− 2.145	− 0.0034		0.3321					4	14.199	− 18.5	0	0.199
0.159								2	11.454	− 18.4	0.11	0.189
0.209	− 0.0016							3	12.542	− 18.0	0.50	0.155
− 2.641	− 0.0037	− 0.751	0.3974					5	14.479	− 16.0	2.54	0.056
− 4.748	− 0.0032	− 61.660	0.6889			8.536		6	16.161	− 15.9	2.59	0.055

For each site and year, HQF follows a curvilinear descending pattern as S increases that is best captured by a Gompertz sigmoidal fit (R^2^ = 0.798, *p* < 0.0001) with the equation:

7 $${\text{HQF}} = {1}.0{116}/({1} + {\text{exp}}( - ({\text{S}} - {3}.{1816})/ - 0.{2782}))$$

**Fig. 8:**
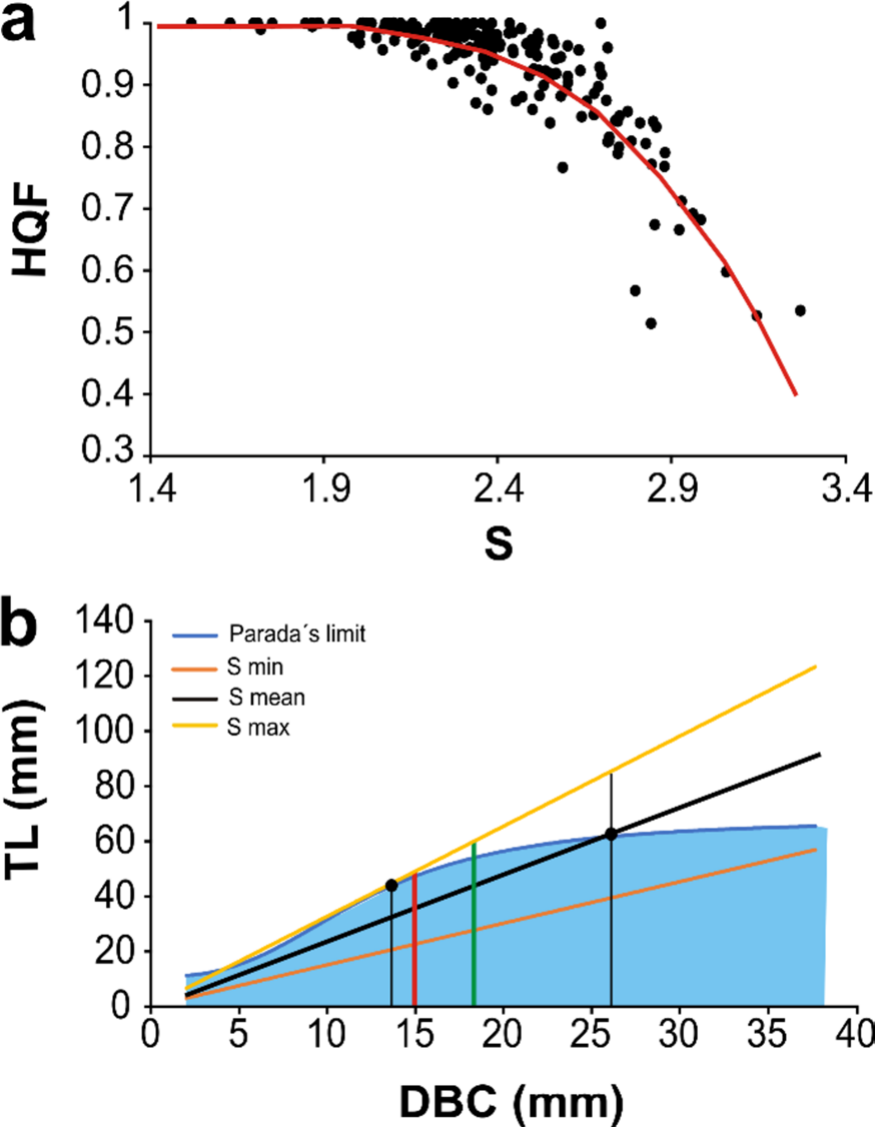
**a** Gompertz sigmoidal fit (red line) between HQF and S. Each point corresponds to a yearly mean within a given site. **b** Parada’s quality limit (blue line) in the relationship between total length (TL) and DBC. The blue surface shows the DBC-TL domain which corresponds to high quality stalked barnacle. DBC-TL relationships corresponding to average (2.37), maximum (3.27) and minimum (1.51) S yearly means for all sites are shown with black, yellow, and orange lines, respectively. The points and their corresponding thin dark vertical lines represent the intersections between Parada’s limit and the Smean and Smax lines. The red and green thick vertical lines represent the minimum legal DBC size of 15 mm and Sestelo’s size of capture of 18.01 mm, respectively

Overall, when S yearly means at a site become greater than 2, then the percentage of high quality *P. pollicipes* within the population decreases from its maximum value of 100% (Fig. 8a).

The DBC vs TL plot allows the comparison between Parada’s quality limit and mean, minimum and maximum S yearly averages from all sites (Fig. 8b). As Parada’s limit is curvilinear, the rate at which maximum TL of good quality specimens increases decreases with DBC, reaching a plateau around 6 cm if DBC overpasses 2.5 cm. Thus, only small animals below the DBC legal limit of 15 mm can be roughly considered of good quality if S reaches our observed population maximum of 3.27. If S is around the mean population average of 2.37, then good quality barnacles can be obtained up to much bigger sizes corresponding to DBC values around 2.6 cm. Finally, if the lowest S mean values of 1.51 are considered instead, then all animals within the observed DBC range may fall within the good quality category.

## Discussion

### Trends in wave climate

Our results from the time series analyses performed on SIMAR wave datasets off the coasts of A Guarda and Baiona point to an increase in both maximum wave heights and their corresponding inshore orbital currents (Fig. 2). These findings are consistent with wave heights increases recorded and forecasted for the Iberian coast as the eastward winter storm track is displaced to the north towards the British Islands by the northward movement of the Azores Anticyclone, a process driven by anthropogenic climate change (Kushnir et al. 1997; Dupuis et al. 2006; Young et al. 2011; Castelle et al. 2018). Under this climatic forcing, the occurrence at western Iberia of winter high swell seas coming from the northward-shifted storm track has increased (Dupuis et al. 2006; Hochet et al. 2021). Nevertheless, when only the peaking wave season is considered, this temporal trends are blurred, especially after 2014, just when most of our stalked barnacle time series started (Table 1). Accordingly, Castelle et al. (2018) observed increments in wintertime wave height, but also in its variability, along time series derived from reanalysis models from 1949 to 2017.

The overall positive trends detected by our GAM analyses are best explained by much clearer increases in summer, during the season with the minimum records in maximum wave height (Fig. 2, see Figure 1 in Supplementary Material). These observations agree with summer increases in swell and wind wave predicted by the ERA-Interim model (Dee et al. 2011) for western Iberia from the 70s to 2013, while such rises were smaller in winter (Semedo et al. 2014). Furthermore, forecasts for the end of the twenty-first century calibrated with the same ERA-Interim model point to increments in significant wave height only in summer (Bricheno and Wolf 2018). These summer trends may be driven by an increased number of hurricanes between June and November, a pattern detected along the eastern U.S. coast from 35 years wave time series recorded at offshore buoys (Komar and Allan 2008). More recently, rises in wind wave heights have been attributed to the proximity of tropical storms off the Portuguese coasts (Oliveira et al. 2020). This hurricane intensification is determined by increased sea surface temperatures in the tropical Atlantic and the eastward extension of the warm water pool where tropical cyclones are generated is reducing their travel distance to Europe (Haarsma et al. 2013; Bricheno and Wolf 2018). Moreover, recent works have shown that the incidence of winter storms passing through western Europe and the strength of the hurricane season are negatively correlated through an El Niño-driven teleconnection (Schemm et al. 2018; Angus and Leckebusch 2020). Thus, summer increases in wave heights could prevail in the future while wintertime increases reverse. Still, although steadily rising, summer orbital currents attain speeds between 4 to 5 m/s, while in winter they range from 5 to 8 m/s (Fig. 2, see Figure 1 in Supplementary Material). Therefore, summer wave climate may not be rough enough to suppose a major selection force for *P. pollicipes* morphological patterns at the population level, at least not in the near future. On the other hand, winter wave conditions will continue to exert the maximum mechanical stress on the barnacles, although the long term evolution of such conditions is quite uncertain.

### Trends in *P. pollicipes* morphometry and quality

In *Pollicipes polymerus* the causes presented to explain phenotypical variation were mainly related to wave exposure and to the relative position of the barnacles on the clump (Barnes and Reese 1960). The barnacles with strong and short peduncles were associated with very wave-exposed locations, while barnacles with an elongated peduncle were associated with more sheltered places (Barnes and Reese 1960). Also, slender individuals were generally located in the center of the barnacles clumps of this species (Chaffee and Lewis 1974). In *Pollicipes pollicipes,* this phenotypical variation was associated with differences in individual density, with locations with higher densities having thinner and longer animals (Cruz 2000). However, this factor might be confounded with other factors such as hydrodynamics or predation (Cruz 2000).

Typically, the spatial planning for the extraction of stalked barnacles along the Galician coast and the Cantabrian Sea has been based on gatherer's perception of resource quality specific for each site and sometimes even for each isolated rock within each site (Molares and Freire 2003; Rivera et al. 2014, 2016a). In more recent works, these quality assessments were correlated successfully with coastal topographic parameters, which suggests that higher qualities are associated to very exposed rocky shores, thus confirming harvesters’ traditional knowledge on the characteristics of the best sites for harvesting (Rivera et al. 2016b; Parada et al. 2012). In fact, in this work we have observed a clear association between our HQF assessments and the quality categorization established by harvesters (Fig. 4b). In parallel to this body of research, Parada et al. (2012) found a non-linear but clearly significant relationship between quality perception and barnacle morphology with the TL-DBC plots. Overall, all these works point towards a determination of organismal shape and in turn quality driven by nearshore hydrodynamics that vary along the coast. If such association is true, then temporal changes in wave climate should also impact barnacle morphometry and quality. In other words, the quality-hydrodynamics relationship in space should also hold in time. We found evidence that this is the case for the coasts of Baiona and A Guarda, as trends in S and HQF corresponded to changes in orbital currents (Figs. 2, 6). In the North America Pacific species of the genus *Pollicipes, P. polymerus,* phenotypical variation was linked to wave exposure inhabiting in wave-exposed locations animals with wide and short peduncles (Barnes and Reese 1960).

Consistently, temporal trends in organismal adaptations to wave temporal shifts have been observed in other rocky shore species. Blue mussels *Mytilus edulis* presented stronger and more abundant byssal threads around the winter storm season along the US east coast (Carrington 2002a, b). However, laboratory experiments with the same species revealed that wave induced flows elicited a decrease in thread production, not an increase (Moeser et al. 2006). Clearer effects were observed in the barnacle *Amphibalanus eburneus* in the Gulf of Mexico*,* which shortened its cirri in less than 2 weeks when placed in a new, rougher environment with faster flows (Reustle et al. 2023). These responses can be species-specific, as along the Chilean coasts where the barnacle *Jehlius cirratus* shows a wide plasticity in cirri morphology with wave stress monthly variations, while the very similar *Notochthamalus scabrosus* does not (López et al. 2010). Regarding overall morphometry, Cruz (2000) found that the rostro carinal length (RC), and in turn DBC, increased 4.3 times faster in wintertime. Furthermore, Pavón (2003) observed faster growth rates under rougher conditions of longer wave periods and stronger winds in Asturias at the central Cantabrian coast. These results suggest that phenotypic plasticity triggered by enhanced wave stress is what drives variations in growth towards relatively wider individuals. The mechanisms behind such an adaptation can be based on epigenetic switching of different developmental pathways during the life of the individual, a process that is known to operate in barnacles (Kaji and Palmer 2017; Marchinko 2003; Neufeld 2012). Direct selection of relatively wider individuals by wave driven mortality acting on elongated barnacles is unlikely, as that would imply that relative width is an inherited character. However, genetic studies did not find distinctive genotypes for different morphs in *P. pollicipes* (Campo 2009; Sousa et al. 2021; Cruz et al. 2022).

### Potential management consequences

Regardless of its plasticity, clear changes in *Pollicipes pollicipes* morphometry do not translate easily to conspicuous changes in quality at the population level, although they do correlate as shown in Fig. 5. This is evidenced by the lack of association between HQF and time (Fig. 4), and the worse fits between orbital currents, site specific topography and HQF compared with those for S (Figs. 6, 7). We believe that it is due to the non-linear link between S and the curvilinear Parada’s quality limit, as the relative length of good quality barnacles decreases as DBC increases. In essence, as animals become elongated from population S means of 1.5 to 3, wider individuals, according to their DBC, fall faster into the bad quality domain than thinner ones (Fig. 8b). However, from S means between 1.5 and 2.4, slight increases in S do not suppose any increase in the probability of getting above the quality limit, not even for the widest individuals (Fig. 8b). On the contrary, in between S values of 2.4 and 3, the same increments in S are translated to exponential drops in quality, especially for wider individuals. This pattern explains the curvilinear relationship between mean S and HQF (Fig. 8a).

Such asymmetric morphometry-quality association may not be trivial and could have consequences for the management of this coastal fishery. Note that the S grand mean for all years and sites is 2.37, quite close to the 2.4 threshold that signals the beginning of fast quality losses (Fig. 8). When inspected closer, for the 200 yearly S means for all sites and years, 117 fall below 2.4 (58.5%) and 83 (41.5%) fall above this limit. This relative skewness towards low S values may be behind the refractory behaviour of HQF to shifts in S, as for the majority of sites and years S falls well below the Parada’s limit regardless of DBC (Fig. 8b). However, if S starts to overpass 2.4 frequently, driven by slower orbital currents below 7 m/s (Fig. 6) produced by wintertime maximum waves shorter than 5 m (Eq. 4), then quality will drop dramatically. Furthermore, in this scenario, thinner barnacles with 2 cm of DBC or below, may be the only ones retaining the good quality category (Fig. 8) and in turn they may be subjected to most of the harvesting efforts. As thinner individuals are also the younger ones (Parada et al. 2012), such a potential shift in the size of collected barnacles could lead to a reduction of the reproductive output at the population level because of the removal of young individuals that have spawned just a few times. The minimum size of sexual maturity, at least in functional females, is around 12 mm of rostro carinal (RC) length in *P. pollicipes* (Cruz and Araújo 1999; Sestelo and Roca-Pardiñas 2007) which yields 9.1 mm in DBC (Parada et al. 2013), well below the legal limit of 15 mm. Usually, such small barnacles lack commercial value (Outerial and Amoedo, personal communication), but preferential collection of valuable high quality barnacles between 1.5 and 2 cm in DBC could be problematic. Even at the optimum size of capture of 21.5 mm in RC (18.01 mm in DBC) which maximizes barnacle growth, yield and reproductive output (Sestelo and Roca-Pardiñas 2007), quality could be bad if the S population mean attains its recorded maximum of 3.27 (Fig. 8b). Nevertheless, this is just a deductive exercise and a DBC potential reduction in collected animals at sites presenting a substantial drop in barnacle quality over time should be confirmed with fish market time series in future studies.

### Coastal topography regulation of the wave-morphology coupling

We found that barnacle location across the intertidal affected overall quality, although slightly. HQF was significantly lower in the mid intertidal with respect to higher and lower heights in the shore (Fig. 4a), but the difference is minimal and only comes significant in our analysis due to the large N used. In southern Portugal, *P. pollicipes* growth rates measured in RC (and in turn DBC) were found to be higher in the lower intertidal than higher in the shore (Cruz 2000; Cruz et al. 2010; Neves 2021), possibly indicating a higher biological performance of animals with increased submersion times, and/or higher wave stress. However, these spatial differences in growth rates across the intertidal were not found in the coasts of Asturias (Pavón 2003). Temporal trends in S at the 3 intertidal levels were also inconsistent and site-specific, as in some locations S remained constant but in others it varied widely (Fig. 3, see Table 1 in Supplementary Material).

Trends in wave patterns at a regional scale may have different effects on barnacle morphometry and quality at a local scale depending on coastal topography. Consistently, the wave forcing-morphometry coupling varied widely among sites (Fig. 6). Thus, the coastal landscape acts as a selective barrier for the impact of wave dynamics on barnacle morphology at the population level. In particular, patterns in S are best explained by wave induced orbital currents at gentle slopes exposed to relatively moderate wave forcing, as revealed by the interactive effect of u and slope on R^2^S (Fig. 7a). Accordingly, gentle slopes are associated with dissipative coastal stretches where wave entrainment is facilitated with respect to more stepper, reflective coasts where waves rebound seawards (Defeo and McLachlan 2011; Morgan et al. 2017; Zhang et al. 2021). In fact, the transport of sediment and planktonic larvae driven by orbital currents across the surf zone is known to be maximised at flat shores (Navarrete et al. 2015; Shanks et al. 2017; Weidberg et al. 2018). On the other hand, the increases in wave period associated with the largest swells are known to increase wave reflection especially at steep shorelines (Muttray et al. 2006; Mahmoudof et al. 2021). This relationship could explain why the interaction between gentle slopes and relatively low orbital currents (and not high) favours the wave-barnacle morphometry coupling.

Regarding barnacle quality, the variability in the orbital current-HQF association was best explained by the relative wave incidence angle with a residual role of the site-specific mean orbital current (Fig. 7b). Overall, this model explains far less variability than the ones for R^2^S (12% vs 50%), but there is a slight reduction of R^2^HQF as the coastline becomes more protected from the incoming waves (Fig. 7b). In agreement with these results, wave power and energy is known to increase at perpendicular shores with respect to wave prevalent direction, as shown by studies on coastal structures and wave energy extraction devices (Denny 1995; Falnes 2007). These effects are effectively translated to the intertidal ecosystems. For instance, wave fetch, that is turn determined by wave incidence angle, was shown to be the main factor structuring intertidal communities along the coasts of Scotland (Burrows et al. 2008). At the species specific level, plastic morphological changes in the kelp *Ecklonia radiata* occur at sites exposed to the wave prevalent direction, but not at sheltered sites (Fowler-Walker et al. 2005).

When planning the relative fishing effort to be carried out at each site within each management plan, the high spatial heterogeneity in the wave climate-barnacle quality coupling has to be taken into account. Thus, in a scenario where winter wave forcing becomes milder, there will be sites refractory to a change in quality, while in others quality will decrease much faster. Given its northward facing coast, Baiona sites would be less prone to wave driven quality shifts, although there is variability between locations. For instance, B03 (Baleal), considered of high quality site in the harvesters’ assessment (Table 1), faces north and presents a relatively high HQF that remains relatively constant regardless of trends in orbital currents. However, just 1 km to the east, B10 (Bombardeira) is more sensitive to shifts in wave climate as it is orientated to the northwest. Its mean HQF is high and it is highly valuated by harvesters (high quality, Table 1). Thus, if available meteorological forecasts point to a decrease in wave height for a given winter, to direct fishing efforts to B03 rather than to B10 would be a safe strategy. Along A Guarda a similar exercise can be done, with G13 (S Carabeleira) showing a mean high HQF that does not change with wave forcing, which suggests that it could be exploited even after a mild winter with relatively small maximum wave heights. On the contrary, with the same wave conditions, barnacle extractions at G15 (S Os Soles) should probably be avoided given the high HQF-orbital current association. Harvesters regard both sites as very low quality ones (Table 1), although our samplings show consistently higher HQF mean values at G13 than at G15 (Fig. 4b). Given this difference in HQF and its refractory behaviour against changing wave climate, G13 should be exploited first.

## Conclusions

In this research work, we have inspected for the first time the trends in *Pollicipes pollicipes* morphometry and market quality in relation to shifts in coastal wave climate for a total of 26 extraction sites at 2 management plans in western Iberia. We found evidence of temporal changes in barnacle quality and, especially, morphometry driven by concurrent shifts in winter wave induced orbital currents. Although correlated, temporal trends in relative length S are not translated to changes in quality of the same magnitude, probably because of the non-linear nature of the Parada’s quality limit relating length and width of the animal. Thus, large increases in mean population S beyond 2.4 are required to drive significant drops in quality, as smaller S values fall always below the Parada’s limit. However, beyond 2.4, only thin individuals in between 15 and 20 mm in DBC would be considered of high quality, which could be of concern for this fishery. This effect occurs due to the non-linear shape of the Parada’s limit because, as DBC increases, only relatively wider animals with lower S values remain as good quality barnacles. As S grand mean for all sites is 2.37, it is possible to overpass the 2.4 threshold if wave heights decrease, which would be translated to rapid decreases in quality. However, because trends in wave climate are decoupled from *P. pollicipes* morphometry at steeper sites covered from the prevalent wave direction, wave climate shifts in the mesoscale will have different effects on the small spatial scale of each extraction site. Thus, coastal topography acts as an effective selective barrier buffering the impact of shifting wave climate on barnacle morphology at least at certain coastal sites. This knowledge could be applied to build barnacle extraction site specific strategies based on yearly wave climate forecasts.

### Supplementary Information

Below is the link to the electronic supplementary material.

Supplementary file1 (DOCX 197 kb)

## Data Availability

The datasets of the current study are available from the corresponding author on reasonable request.

## References

[CR1] Álvarez I, Ospina-Álvarez N, Pazos Y, DeCastro M, Bernardez P, Campos MJ, Gomez-Gesteira JL, Alvarez-Ossorio MT, Varela M, Gomez-Gesteira M, Prego R (2009). A winter upwelling event in the Northern Galician Rias: frequency and oceanographic implications. Estuar Coast Shelf Sci.

[CR2] Angus M, Leckebusch GC (2020). On the dependency of Atlantic hurricane and European windstorm hazards. Geophys Res Lett.

[CR3] Bakun A, Nelson CS (1991). The seasonal cycle of wind-stress curl in subtropical eastern boundary current regions. J Phys Oceanogr.

[CR102] Barnes H, Reese ES (1960) The behaviour of the stalked intertidal barnacle Pollicipes polymerus JB Sowerby, with special reference to its ecology and distribution. J Anim Ecol 169–185

[CR4] Barnes M (1996). Pedunculate cirripedes of the genus Pollicipes. Oceanogr Mar Biol.

[CR100] Bartoń K (2022) MuMIn: Multi-Model Inference. R package version 1.47.1. https://CRAN.R-project.org/package=MuMIn

[CR5] Bindoff NL, Willebrand J, Artale V, Cazenave A, Gregory JM, Gulev S, Hanawa K, Le Quere C, Levitus S, Nojiri Y, Shum CK, Talley LD, Unnikrishnan AS, Josey SA, Tamisiea M, Tsimplis M, Woodworth P (2007). Observations: oceanic climate change and sea level.

[CR6] Blanton JO, Atkinson LP, Fernández-de-Castillejo F, Lavin-Montero A (1984). Coastal upwelling off the Rias Bajas, Galicia, northwest Spain I: hydrographic studies. Rapp P v Reun Cons Int Explor Mer.

[CR7] Borja A, Liria P, Muxika I, Bald J (2006). Relationships between wave exposure and biomass of the goose barnacle (*Pollicipes pollicipes*, Gmelin, 1790) in the Gaztelugatxe Marine Reserve (Basque Country, northern Spain). ICES J Mar Sci.

[CR8] Boukaici M, Bergayou H, Kaaya A (2012). *Pollicipes pollicipes* (Gmelin, 1789) (Cirripède, Lepadomorphe): étude de la croissance et de la dynamique des populations dans la région de Mirleft (sud ouest Marocain). Crustaceana.

[CR9] Bricheno LM, Wolf J (2018). Future wave conditions of Europe, in response to high-end climate change scenarios. J Geophys Res Oceans.

[CR10] Burrows MT, Harvey R, Robb L (2008). Wave exposure indices from digital coastlines and the prediction of rocky shore community structure. Mar Ecol Prog Ser.

[CR11] Campo D, Molares J, Garcia L, Fernandez-Rueda P, Garcia-Gonzalez C, Garcia-Vazquez E (2010). Phylogeography of the European stalked barnacle (*Pollicipes pollicipes*): identification of glacial refugia. Mar Biol.

[CR12] Campo D (2009) Micro-y macroevolución en organismos marinos. Dissertation, Universidad de Oviedo

[CR13] Carrington E (2002). Seasonal variation in the attachment strength of blue mussels: causes and consequences. Limnol Oceanogr.

[CR14] Carrington E (2002). The ecomechanics of mussel attachment: from molecules to ecosystems. Integr Comp Biol.

[CR15] Castelle B, Dodet G, Masselink G, Scott T (2017). A new climate index controlling winter wave activity along the Atlantic coast of Europe: the West Europe Pressure Anomaly. Geophys Res Lett.

[CR16] Castelle B, Dodet G, Masselink G, Scott T (2018). Increased winter-mean wave height, variability, and periodicity in the Northeast Atlantic over 1949–2017. Geophys Res Lett.

[CR103] Chaffee J, Lewis CA (1974). Pedunculate barnacle stalk growth. J Exp Mar Biol Ecol.

[CR17] Chen C (2000). Generalized additive mixed models. Commun Stat Theory Methods.

[CR18] Cruz T (2000) Biologia e ecologia do percebe *Pollicipes pollicipes* (Gmelin, 1790), no litoral sudoeste portugês. Dissertation, Universidade de Evora (Portugal)

[CR19] Cruz T, Araújo J (1999). Reproductive patterns of *Pollicipes pollicipes* (Cirripedia: Scalpellomorpha) on the southwestern coast of Portugal. J Crust Biol.

[CR20] Cruz T, Castro JJ, Hawkins SJ (2010). Recruitment, growth and population size structure of *Pollicipes pollicipes* in SW Portugal. J Exp Mar Biol Ecol.

[CR21] Cruz T, Jacinto D, Fernandes JN, Seabra MI, Van Syoc RJ, Power AM, Macho G, Sousa A, Castro JJ, Hawkins SJ (2022). Pedunculate cirripedes of the genus Pollicipes: 25 years after Margaret Barnes’ review. Oceanogr Mar Biol Annu Rev.

[CR22] Dee DP, Uppala SM, Simmons AJ, Berrisford P, Poli P, Kobayashi S (2011). The ERA-Interim reanalysis: configuration and performance of the data assimilation system. Q J R Meteorol.

[CR23] Defeo O, McLachlan A (2011). Coupling between macrofauna community structure and beach type: a deconstructive meta-analysis. Mar Ecol Prog Ser.

[CR24] Denny M (1988). Biology and the mechanics of the wave-swept environment.

[CR25] Denny M (1995). Predicting physical disturbance: mechanistic approaches to the study of survivorship on wave-swept shores. Ecol Monogr.

[CR26] Denny M (2014). Biology and the mechanics of the wave-swept environment.

[CR27] Denny MW, Gaylord B (2010). Marine ecomechanics. Annu Rev Mar Sci.

[CR28] Denny MW, Hunt LJ, Miller LP, Harley CD (2009). On the prediction of extreme ecological events. Ecol Monogr.

[CR29] Dodet G, Bertin X, Taborda R (2010). Wave climate variability in the North-East Atlantic Ocean over the last six decades. Ocean Model.

[CR30] Draper L (1986) Wave measurements at North Atlantic Weather stations “India” and “Juliett” in the 1970s (India 2 and Juliett 2). Oceanology: proceedings of an international conference, Brighton

[CR31] Dupuis H, Michel D, Sottolichio A (2006). Wave climate evolution in the Bay of Biscay over two decades. J Mar Syst.

[CR32] Falnes J (2007). A review of wave-energy extraction. Mar Struct.

[CR33] Feser F, Barcikowska M, Krueger O, Schenk F, Weisse R, Xia L (2015). Storminess over the North Atlantic and northwestern Europe: a review. Q J R Meteorol Soc.

[CR34] Fowler-Walker MJ, Connell SD, Gillanders BM (2005). To what extent do geographic and associated environmental variables correlate with kelp morphology across temperate Australia?. Mar Freshw Res.

[CR35] GEBCO Compilation Group (2020) GEBCO 2020 Grid. 10.5285/a29c5465-b138-234d-e053-6c86abc040b9

[CR36] Gillett NP, Graf HF, Osborn TJ (2003). Climate change and the North Atlantic oscillation. Geophys Monogr Ser.

[CR37] Haarsma RJ, Hazeleger W, Severijns C, De Vries H, Sterl A, Bintanja R, van Oldenborgh GJ, van den Brink HW (2013). More hurricanes to hit western Europe due to global warming. Geophys Res Lett.

[CR38] Hewitt JE, Ellis JI, Thrush S (2015). Multiple stressors, non linear effects and the implications of climate change impacts on marine coastal ecosystems. Glob Change Biol.

[CR40] Hochet A, Dodet G, Ardhuin F, Hemer M, Young I (2021). Sea state decadal variability in the North Atlantic: a review. Clim.

[CR41] Höfer J, Muñiz C, Weidberg N, García-Flórez L, Acuña JL (2017). High densities of stalked barnacle larvae (*Pollicipes pollicipes*) inside a river plume. J Plankton Res.

[CR42] Hurrell JW (1995). Decadal trends in the North Atlantic Oscillation: regional temperatures and precipitation. Science.

[CR43] IPCC (2022) Special report of the IPCC: the ocean and cryosphere in a changing climate. In: Pörtner H-O, Roberts DC, Masson-Delmotte V, Zhai P, Tignor M, Poloczanska E, Mintenbeck K, Alegría A, Nicolai M, Okem A, Petzold J, Rama B, Weyer NM (eds). Cambridge University Press, Cambridge, United Kingdom and New York, NY, USA

[CR44] Kaji T, Palmer AR (2017). How reversible is development? Contrast between developmentally plastic gain and loss of segments in barnacle feeding legs. Evolution.

[CR45] Kamphuis JW, Davies MH, Nairn RB, Sayao OJ (1986). Calculation of littoral sand transport rate. Coast Eng.

[CR46] Komar PD, Allan JC (2008). Increasing hurricane-generated wave heights along the US East Coast and their climate controls. J Coast Res.

[CR47] Kushnir Y, Cardone VJ, Cane M (1995) Link between North Atlantic climate variability of surface wave height and sea level pressure. In: 4th international workshop on Wave Hindcasting and Forecasting, Environment Canada, Banff, Alberta, Canada

[CR48] Kushnir Y, Cardone VJ, Greenwood JG, Cane MA (1997). The recent increase in North Atlantic wave heights. J Clim.

[CR49] Lima FP, Wethey DS (2012). Three decades of high-resolution coastal sea surface temperatures reveal more than warming. Nat Commun.

[CR50] López BA, Ramírez RP, Guaitro SY, López DA (2010). Interspecific differences in the phenotypic plasticity of intertidal barnacles in response to habitat changes. J Crust Biol.

[CR99] Lund U, Agostinelli C (2001) CircStats: Circular Statistics, from “Topics in Circular Statistics”. R package version 0.2-6. https://CRAN.R-project.org/package=CircStats

[CR51] Macho G, Molares J, Vázquez E (2005). Timing of larval release by three barnacles from the NW Iberian Peninsula. Mar Ecol Prog Ser.

[CR52] Madin JS (2005). Mechanical limitations of reef corals during hydrodynamic disturbances. Coral Reefs.

[CR53] Mahmoudof SM, Eyhavand-Koohzadi A, Bagheri M (2021). Field study of wave reflection from permeable rubble mound breakwater of Chabahar Port. Appl Ocean Res.

[CR54] Marchinko KB (2003). Dramatic phenotypic plasticity in barnacle legs (*Balanus glandula* Darwin): magnitude, age dependence, and speed of response. Evolution.

[CR55] McClain CR, Chao SY, Atkinson LP, Blanton JO, De Castillejo F (1986). Wind-driven upwelling in the vicinity of Cape Finisterre, Spain. J Geophys Res Oceans.

[CR101] McFadden D (1974). The measurement of urban travel demand. J Public Econ.

[CR56] Meucci A, Young IR, Hemer M, Kirezci E, Ranasinghe R (2020). Projected 21st century changes in extreme wind-wave events. Sci Adv.

[CR57] Moeser GM, Leba H, Carrington E (2006). Seasonal influence of wave action on thread production in *Mytilus edulis*. J Exp Biol.

[CR58] Molares J, Freire J (2003). Development and perspectives for community-based management of the goose barnacle (*Pollicipes pollicipes*) fisheries in Galicia (NW Spain). Fish Res.

[CR59] Molares J, Tilves F, Pascual C (1994). Larval development of the pedunculate barnacle *Pollicipes cornucopia* (Cirripedia: Scalpellomorpha) reared in the laboratory. Mar Biol.

[CR60] Morgan SG, Shanks AL, MacMahan J, Reniers AJHM, Griesemer CD, Jarvis M, Fujimura AG (2017). Surf zones regulate larval supply and zooplankton subsidies to nearshore communities. Limnol Oceanogr.

[CR61] Muttray M, Oumeraci H, ten Oever E (2006) Wave reflection and wave run-up at rubble mound breakwaters. In: Conference paper 30th ICCE, pp 4313–4324. 10.1142/9789812709554_0362

[CR62] Navarrete SA, Largier JL, Vera G, Tapia FJ, Parragué M, Ramos E, Shinen JL, Stuardo CA, Wieters EA (2015). Tumbling under the surf: wave-modulated settlement of intertidal mussels and the continuous settlement-relocation model. Mar Ecol Prog Ser.

[CR63] Neu HJA (1984). Interannual variations and longer-term changes in the sea state of the North Atlantic from 1970 to 1982. J Geophys Res Oceans.

[CR104] Neufeld CJ (2012). Barnacle appendage plasticity: asymmetrical response time-lags, developmental mechanics and seasonal variation. J Exp Mar Biol Ecol.

[CR64] Neves FCFD (2021) Biologia e conservação do percebe (*Pollicipes pollicipes*) na Reserva Natural das Berlengas. Dissertation, Universidade de Évora

[CR65] Oliveira TC, Cagnin E, Silva PA (2020). Wind-waves in the coast of mainland Portugal induced by post-tropical storms. Ocean Eng.

[CR66] Orfila A, Alvarez A, Tintoré J, Jordi A, Basterretxea G (2005). Climate teleconnections at monthly time scales in the Ligurian Sea inferred from satellite data. Prog Oceanogr.

[CR67] Parada JM, Outeiral R, Iglesias E, Molares J (2012). Assessment of goose barnacle (*Pollicipes pollicipes* Gmelin, 1789) stocks in management plans: design of a sampling program based on the harvesters' experience. ICES J Mar Sci.

[CR68] Parada JM, Iglesias E, Outeiral R, Molares J (2013). Diameter of the base of the capitulum as a biometric variable of the goose barnacle *Pollicipes pollicipes* (Gmelin, 1789)(Cirripedia, Lepadomorpha). Crustaceana.

[CR69] Pavón MC (2003) Biología y variables poblacionales del percebe, *Pollicipes pollicipes* (Gmelin, 1790) en Asturias. Dissertation, Universidad de Oviedo

[CR70] Quinteiro J, Rodríguez-Castro J, Rey-Méndez M (2007). Population genetic structure of the stalked barnacle *Pollicipes pollicipes* (Gmelin, 1789) in the northeastern Atlantic: influence of coastal currents and mesoscale hydrographic structures. Mar Biol.

[CR71] R Core Team (2022) R: a language and environment for statistical computing. R Foundation for Statistical Computing, Vienna, Austria. https://www.R-project.org/

[CR72] Reguero BG, Losada IJ, Méndez FJ (2019). A recent increase in global wave power as a consequence of oceanic warming. Nat Commun.

[CR73] Reustle JW, Belgrad BA, McKee A, Smee DL (2023). Barnacles as biological flow indicators. PeerJ.

[CR74] Rivera A, Weidberg N, Pardinas AF, Gonzalez-Gil R, García-Flórez L, Acuña JL (2013). Role of upwelling on larval dispersal and productivity of gooseneck barnacle populations in the Cantabrian Sea: management implications. PLoS ONE.

[CR75] Rivera A, Gelcich S, García-Florez L, Alcázar JL, Acuña JL (2014). Co-management in Europe: insights from the gooseneck barnacle fishery in Asturias, Spain. Mar Policy.

[CR76] Rivera A, Gelcich S, García-Flórez L, Acuña JL (2016). Assessing the sustainability and adaptive capacity of the gooseneck barnacle co-management system in Asturias. N Spain Ambio.

[CR77] Rivera A, Gelcich S, Garcia-Florez L, Acuña JL (2016). Incorporating landscape metrics into invertebrate fisheries management: case study of the gooseneck barnacle in Asturias (N. Spain). ICES J Mar Sci.

[CR78] Román S, Weidberg N, Muniz C, Aguion A, Vázquez S, Santiago J, Seoane P, Barreiro B, Outeiral R, Villegas-Rios D, Macho G (2022). Mesoscale patterns in barnacle reproduction are mediated by upwelling driven thermal variability. Mar Ecol Prog Ser.

[CR79] Schemm S, Rivière G, Ciasto LM, Li C (2018). Extratropical cyclogenesis changes in connection with tropospheric ENSO teleconnections to the North Atlantic: role of stationary and transient waves. J Atmos Sci.

[CR80] Semedo A, Cabrera Bermejo H, Martinho P, Bernardino M, Guedes Soares C (2014) Recent trends in of the North Atlantic wave heights from ERA-Interim. EMS conference paper

[CR81] Sestelo M, Roca-Pardiñas J (2007) Length-weight relationship of *Pollicipes pollicipes* (Gmelin, 1789) on the Atlantic coast of Galicia (NW Spain). Some aspects of its biology and management. Discussion papers in statistics and operation research. Report, 10, pp 1–26

[CR82] Shanks AL, MacMahan J, Morgan SG, Reniers JHM (2017). Alongshore variation in barnacle populations is determined by surf zone hydrodynamics. Ecol Monogr.

[CR83] Shimura T, Mori N, Mase H (2013). Ocean waves and teleconnection patterns in the Northern Hemisphere. J Clim.

[CR84] Sierra JP, Casas-Prat M (2014). Analysis of potential impacts on coastal areas due to changes in wave conditions. Clim Change.

[CR85] Simpson GL (2018). Modelling palaeoecological time series using generalised additive models. Front Ecol Evol.

[CR86] Sousa A, Jacinto D, Penteado N, Martins P, Fernandes J, Silva T, Castro JJ, Cruz T (2013). Patterns of distribution and abundance of the stalked barnacle (*Pollicipes pollicipes*) in the central and southwest coast of continental Portugal. J Sea Res.

[CR87] Sousa MC, Ribeiro A, Des M, Gomez-Gesteira M, deCastro M, Dias JM (2019). NW Iberian Peninsula coastal upwelling future weakening: competition between wind intensification and surface heating. Sci Total Environ.

[CR88] Sousa A, Vázquez E, Macho G, Acuña JL, Cruz T, Morán P (2021) The stalked barnacle *Pollicipes pollicipes* elongated morphology: no evidence for epigenetic variation. In: Arias A et al (eds) Proceedings of the XVII international symposium on oceanography of the bay of biscay (ISOBAY 17). University of Oviedo, Oviedo, Spain, 92 pp

[CR89] Walden H, Hogben N, Burkhart MD, Dorrestein R, Warnsink WH, Yamanouchi Y (1970) Long term variability. 4th International Ship Structures Congress, Tokyo, Report of Committee, vol 1, pp 49–59

[CR90] Weidberg N, Bularz B, López-Rodríguez S, Navarrete SA (2018). Wave-modulation of mussel daily settlement at contrasting rocky shores in central Chile: topographic regulation of transport mechanisms in the surf zone. Mar Ecol Prog Ser.

[CR91] Weidberg N, Ospina-Alvarez A, Bonicelli J, Barahona M, Aiken CM, Broitman BR, Navarrete SA (2020). Spatial shifts in productivity of the coastal ocean over the past two decades induced by migration of the Pacific Anticyclone and Bakun's effect in the Humboldt Upwelling Ecosystem. Glob Planet Change.

[CR92] Wolanski E, Spagnol S (2000). Sticky waters in the Great Barrier Reef. Estuar Coast Shelf Sci.

[CR93] Wong PP, Losada IJ, Gattuso JP, Hinkel J, Khattabi A, McInnes KL et al (2014) Coastal systems and low-lying areas. Climate change 2014: climate change 2014: impacts, adaptation, and vulnerability. Part B: regional aspects. Contribution of working group II to the fifth assessment report of the intergovernmental panel on climate change. Cambridge University Press, Cambridge, pp 361–409

[CR94] Wood S (2006). Generalized additive models: an introduction with R.

[CR95] Woolf DK, Challenor PG, Cotton PD (2002). Variability and predictability of the North Atlantic wave climate. J Geophys Res Oceans.

[CR96] Wooster WS, Bakun A, McLain DR (1976). Seasonal upwelling cycle along the eastern boundary of the North Atlantic. J Mar Sci.

[CR97] Young IR, Zieger S, Babanin AV (2011). Global trends in wind speed and wave height. Science.

[CR98] Zhang C, Li Y, Zheng J, Xie M, Shi J, Wang G (2021). Parametric modelling of nearshore wave reflection. Coast Eng.

