# Supplementary material for: Influence of a changing wave climate on the quality and morphometry of the stalked barnacle Pollicipes pollicipes (Gmelin, 1789), along the coasts of NW Iberia
Source: Rev Fish Biol Fish. 2024 Mar 14;34(2):781–804. doi: 10.1007/s11160-024-09838-2 (PMC11093743; doi:10.1007/s11160-024-09838-2)
Supplement: Supplementary file 1 — Supplementary file1 (DOCX 197 kb) [file 11160_2024_9838_MOESM1_ESM.docx]

**Appendix**





**Figure 1.** Trends in summer inshore orbital currents from the SIMAR time series at points 3014003 (A), 3014002 (B) and 3014000 (C).

**Table 1.** Results of the best GLMM with S as dependent variable. ***: p<0.001; **: 0.001<p<0.01; *:0.01<p<0.05; . : 0.05<p<0.1 (marginally significant); blank: non significant. Only the second order interaction Time:Intertidal level:Site is shown as it became significant, thus neglecting the main effects of each individual factor and their first order interactions.

| Parameter | Estimate | Std. Error | t value | p value | Significance |
| --- | --- | --- | --- | --- | --- |
| (Intercept) | 5.12E-02 | 3.73E-01 | 0.137 | 0.890964 |  |
| Time:Mid intertidal:G02 | 7.42E-05 | 1.81E-05 | 4.099 | 4.16E-05 | *** |
| Time:High intertidal:G02 | 1.52E-05 | 1.82E-05 | 0.836 | 0.403109 |  |
| Time:Mid intertidal:G03 | -2.24E-05 | 1.88E-05 | -1.196 | 0.231871 |  |
| Time:High intertidal:G03 | 4.41E-05 | 1.86E-05 | 2.364 | 0.0181 | * |
| Time:Mid intertidal:G04 | -3.28E-07 | 2.18E-05 | -0.015 | 0.988001 |  |
| Time:High intertidal:G04 | 3.41E-06 | 2.28E-05 | 0.15 | 0.881141 |  |
| Time:Mid intertidal:G05 | -2.18E-05 | 2.27E-05 | -0.962 | 0.335841 |  |
| Time:High intertidal:G05 | 2.42E-05 | 2.25E-05 | 1.074 | 0.282719 |  |
| Time:Mid intertidal:G06 | -2.22E-05 | 2.23E-05 | -0.996 | 0.319458 |  |
| Time:High intertidal:G06 | 5.72E-05 | 2.23E-05 | 2.57 | 0.010183 | * |
| Time:Mid intertidal:G07 | -5.18E-06 | 2.26E-05 | -0.229 | 0.81902 |  |
| Time:High intertidal:G07 | -1.69E-05 | 2.28E-05 | -0.741 | 0.458487 |  |
| Time:Mid intertidal:G08 | 4.04E-05 | 2.23E-05 | 1.809 | 0.070534 | . |
| Time:High intertidal:G08 | 3.66E-05 | 2.20E-05 | 1.661 | 0.096698 | . |
| Time:Mid intertidal:G09 | 1.00E-04 | 3.28E-05 | 3.053 | 0.002268 | ** |
| Time:High intertidal:G09 | 2.33E-06 | 3.20E-05 | 0.073 | 0.941947 |  |
| Time:Mid intertidal:G10 | 1.14E-05 | 2.00E-05 | 0.573 | 0.566766 |  |
| Time:High intertidal:G10 | -6.59E-05 | 1.97E-05 | -3.354 | 0.000798 | *** |
| Time:Mid intertidal:G11 | 6.83E-05 | 3.33E-05 | 2.053 | 0.040041 | * |
| Time:High intertidal:G11 | 1.21E-04 | 3.31E-05 | 3.645 | 0.000268 | *** |
| Time:Mid intertidal:G12 | -1.00E-04 | 3.14E-05 | -3.188 | 0.001434 | ** |
| Time:High intertidal:G12 | -7.05E-05 | 3.08E-05 | -2.293 | 0.021847 | * |
| Time:Mid intertidal:G13 | -1.19E-05 | 2.66E-05 | -0.448 | 0.654233 |  |
| Time:High intertidal:G13 | 8.59E-06 | 2.70E-05 | 0.318 | 0.75066 |  |
| Time:Mid intertidal:G14 | 1.03E-05 | 1.99E-05 | 0.518 | 0.604785 |  |
| Time:High intertidal:G14 | -1.01E-05 | 2.00E-05 | -0.505 | 0.613847 |  |
| Time:Mid intertidal:G15 | 2.30E-05 | 1.98E-05 | 1.164 | 0.244612 |  |
| Time:High intertidal:G15 | 4.55E-05 | 2.00E-05 | 2.273 | 0.023064 | * |
| Time:Mid intertidal:G16 | -4.48E-06 | 1.81E-05 | -0.247 | 0.804825 |  |
| Time:High intertidal:G16 | 5.78E-05 | 1.83E-05 | 3.163 | 0.001565 | ** |
| Time:Mid intertidal:B01 | 5.36E-05 | 3.54E-05 | 1.515 | 0.129769 |  |
| Time:High intertidal:B01 | 9.62E-05 | 3.24E-05 | 2.972 | 0.00296 | ** |
| Time:Mid intertidal:B02 | 3.21E-05 | 3.30E-05 | 0.972 | 0.330976 |  |
| Time:High intertidal:B02 | 8.69E-05 | 3.23E-05 | 2.693 | 0.007082 | ** |
| Time:Mid intertidal:B03 | -8.25E-06 | 2.85E-05 | -0.289 | 0.772309 |  |
| Time:High intertidal:B03 | -3.03E-05 | 2.78E-05 | -1.093 | 0.274338 |  |
| Time:Mid intertidal:B04 | -1.21E-04 | 3.12E-05 | -3.882 | 0.000104 | *** |
| Time:High intertidal:B04 | -6.18E-05 | 3.04E-05 | -2.036 | 0.04173 | * |
| Time:Mid intertidal:B05 | -1.26E-05 | 2.66E-05 | -0.472 | 0.636702 |  |
| Time:High intertidal:B05 | 4.88E-06 | 2.66E-05 | 0.184 | 0.854211 |  |
| Time:Mid intertidal:B06 | 4.25E-05 | 2.67E-05 | 1.593 | 0.111139 |  |
| Time:High intertidal:B06 | 1.26E-04 | 2.68E-05 | 4.695 | 2.68E-06 | *** |
| Time:Mid intertidal:B07 | 8.95E-05 | 2.81E-05 | 3.185 | 0.001448 | ** |
| Time:High intertidal:B07 | 7.35E-05 | 2.84E-05 | 2.59 | 0.009613 | ** |
| Time:Mid intertidal:B08 | 4.45E-05 | 2.84E-05 | 1.566 | 0.117306 |  |
| Time:High intertidal:B08 | 4.15E-06 | 2.84E-05 | 0.146 | 0.883781 |  |
| Time:Mid intertidal:B09 | 1.16E-04 | 2.79E-05 | 4.145 | 3.41E-05 | *** |
| Time:High intertidal:B09 | 7.86E-05 | 2.80E-05 | 2.811 | 0.004949 | ** |
| Time:Mid intertidal:B10 | 2.01E-06 | 2.77E-05 | 0.073 | 0.942114 |  |
| Time:High intertidal:B10 | 2.61E-06 | 2.74E-05 | 0.095 | 0.924199 |  |


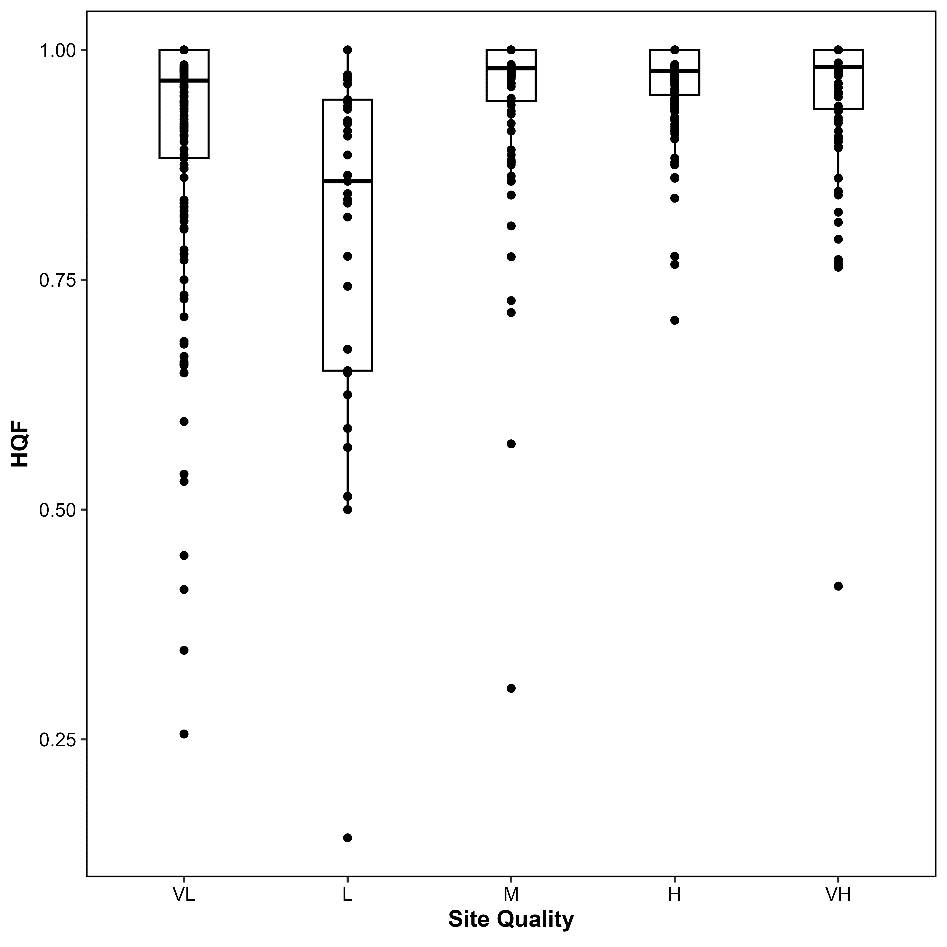


**Figure 2.** Boxplots of High Quality Fraction (HQF) between the different Sites Quality. The black points correspond to each yearly sampling, the black lines show the median and the boxes represent the interquartile range. Quality index of each site: VH=Very high, H=High, M=Medium, L=Low, VL=Very low.

**Table 2.** Results of the GLMM test with High Quality Fraction (HQF) as dependent variable for comparison between quality categories (VL=Very Low; L=Low; M=Medium; H=High; VH=Very High). ***: p<0.001; **: 0.001<p<0.01; *:0.01<p<0.05; . : 0.05<p<0.1 (marginally significant); blank: non significant.

| Quality Category | Estimate | Std. Error | t value | p value | Significance |
| --- | --- | --- | --- | --- | --- |
| Intercept | -0.092713 | 0.008988 | -10.315 | <2E-16 | *** |
| L | -0.135581 | 0.019964 | -6.791 | 2.71E-11 | *** |
| M | 0.039425 | 0.015481 | 2.547 | 0.01113 | * |
| H | 0.042176 | 0.012607 | 4.672 | 3.69E-06 | *** |
| VH | 0.042176 | 0.014820 | 2.846 | 0.00458 | ** |

**Table 3.** Results of the best GLMM with HQF as dependent variable. ***: p<0.001; **: 0.001<p<0.01; *:0.01<p<0.05; . : 0.05<p<0.1 (marginally significant); blank: non significant.

| Parameter | Estimate | Std. Error | t value | p value | Significance |
| --- | --- | --- | --- | --- | --- |
| (Intercept) | -0.0242066 | 0.0221511 | -1.093 | 0.274946 |  |
| Mid intertidal | 0.0028314 | 0.0115948 | 0.244 | 0.80717 |  |
| High intertidal | 0.0299067 | 0.0115811 | 2.582 | 0.01006 | * |
| G02 | -0.0001798 | 0.0298627 | -0.006 | 0.995198 |  |
| G03 | -0.0145265 | 0.0298627 | -0.486 | 0.626841 |  |
| G04 | -0.0007331 | 0.0316742 | -0.023 | 0.981543 |  |
| G05 | -0.0003422 | 0.0316742 | -0.011 | 0.991383 |  |
| G06 | -0.0435274 | 0.0316742 | -1.374 | 0.169913 |  |
| G07 | 0.0016402 | 0.0316742 | 0.052 | 0.95872 |  |
| G08 | -0.2636047 | 0.0316742 | -8.322 | 6.40E-16 | *** |
| G09 | -0.1155262 | 0.0344825 | -3.35 | 0.000861 | *** |
| G10 | -0.2359365 | 0.0306811 | -7.69 | 6.49E-14 | *** |
| G11 | -0.1845289 | 0.0344825 | -5.351 | 1.27E-07 | *** |
| G12 | -0.1494436 | 0.0344825 | -4.334 | 1.73E-05 | *** |
| G13 | -0.0221164 | 0.0329071 | -0.672 | 0.501803 |  |
| G14 | -0.058816 | 0.0306811 | -1.917 | 0.055736 |  |
| G15 | -0.1054546 | 0.0306811 | -3.437 | 0.000631 | *** |
| G16 | -0.0724099 | 0.0298627 | -2.425 | 0.015628 | * |
| B01 | -0.0031509 | 0.0339125 | -0.093 | 0.926005 |  |
| B02 | -0.0138819 | 0.0344825 | -0.403 | 0.687411 |  |
| B03 | -0.04867 | 0.0329071 | -1.479 | 0.13969 |  |
| B04 | -0.0323958 | 0.0344825 | -0.939 | 0.34788 |  |
| B05 | -0.0205315 | 0.0329071 | -0.624 | 0.532928 |  |
| B06 | -0.0841421 | 0.0329071 | -2.557 | 0.010817 | * |
| B07 | -0.0145763 | 0.0329071 | -0.443 | 0.657967 |  |
| B08 | -0.0397956 | 0.0329071 | -1.209 | 0.227037 |  |
| B09 | -0.0344588 | 0.0329071 | -1.047 | 0.295473 |  |
| B10 | -0.0230114 | 0.0329071 | -0.699 | 0.484661 |  |

**Table 4.** Details of the best model resulting from the GLMM analyses for the degree of coupling between S and µ (R^2^S) with relative incidence angle (α), average orbital current (̄$\bar{u}$) and coastal slope as predictors

|  | **Estimate** | **Std. Error** | **t value** | **Pr(>\|t\|)** |  |
| --- | --- | --- | --- | --- | --- |
| (Intercept) | -9.631 | 2.103 | -4.58 | 0.00015 | *** |
| SLOPE | -144.293 | 42.149 | -3.42 | 0.00243 | ** |
| $\bar{u}$ | 1.386 | 0.290 | 4.78 | 0.00009 | *** |
| SLOPE : $\bar{u}$ | 20.576 | 5.895 | 3.49 | 0.00207 | ** |

**Table 5.** Details of the best model resulting from the GLMM analyses for the degree of coupling between HQF and µ (R^2^HQF) with relative incidence angle (α), average orbital current (µ) and coastal slope as predictors.

|  | **Estimate** | **Std. Error** | **t value** | **Pr(>\|t\|)** |  |
| --- | --- | --- | --- | --- | --- |
| (Intercept) | -2.144746 | 1.33171 | -1.611 | 0.1209 |  |
| α | -0.003366 | 0.001456 | -2.312 | 0.0301 | * |
| $\bar{u}$ | 0.332115 | 0.187846 | 1.768 | 0.0903 | . |
